# Evolutionary‐Distinct Viral Proteins Subvert Rice Broad‐Spectrum Antiviral Immunity Mediated by the RAV15‐MYC2 Module

**DOI:** 10.1002/advs.202412835

**Published:** 2025-02-04

**Authors:** Hehong Zhang, Chaorui Huang, Chenfei Gao, Wenkai Yan, Weiqi Song, Xiaodi Hu, Lulu Li, Zhongyan Wei, Yanjun Li, Jianping Chen, Zongtao Sun

**Affiliations:** ^1^ State Key Laboratory for Quality and Safety of Agro‐Products Key Laboratory of Biotechnology in Plant Protection of MARA Zhejiang Key Laboratory of Green Plant Protection Institute of Plant Virology Ningbo University Ningbo 315211 China

**Keywords:** antiviral immunity, jasmonic acid, OsRAV15 transcription factor, rice viruses, viral proteins

## Abstract

A variety of plant viruses employ different virulence strategies to achieve successful infection, resulting in abnormal plant development. However, the common pathogenicity of distinct viruses has rarely been studied. Here, it is shown that a plant‐specific RAV‐type transcription factor, OsRAV15, is specifically targeted by several distinct viral proteins for facilitating viral infection. OsRAV15 is found to activate jasmonic acid (JA)‐mediated broad‐spectrum antiviral immunity by physically associating with JA signaling essential components OsMYC2‐OsJAZ complex. To facilitate viral infection, evolutionarily distinct viral proteins encoded by diverse rice viruses generally disrupt the OsRAV15‐OsMYC2 complex to attenuate activation of JA signaling. Together, the results reveal a common counter‐defense strategy used by different viruses to suppress the OsRAV15‐OsMYC2 module that plays a vital role in fine‐tuning JA‐mediated antiviral defense.

## Graphical abstract

Diverse viruses employ various pathogenicity strategies to inhibit host antiviral defense for successful infection. However, the pathogenic mechanisms conserved among different viruses have rarely been studied. This discovery unveils a broad‐spectrum counter‐defense strategy across evolutionarily distinct viral proteins, highlighting a new strategy for breeding disease‐resistant crops by engineering the OsRAV15‐OsMYC2 module.

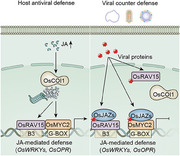

## 1 Introduction

As a globally important food crop, rice is frequently attacked by various viruses, leading to severe yield loss. Among the various viruses infecting rice, rice stripe virus (RSV), Southern rice black‐streaked dwarf virus (SRBSDV), and the newly emerged rice stripe mosaic virus (RSMV) have attracted great attention because they have significant impacts on the yield and quality of crops in East Asia. RSV (*Tenuivirus oryzaclavatae*; *Phenuiviridae*) has a single‐stranded RNA (ssRNA) genome of four components encoding a total of seven proteins. RSV infection causes obvious chlorosis, weakness, and necrosis in emerging leaves. SRBSDV belongs to the genus *Fijivirus* (family *Spinareoviridae*) and causes similar dwarfing symptoms.^[^ ^1^, ^2^ ^]^ The SRBSDV genome has ten segments of double‐stranded RNA (dsRNA). RSMV (*Cytorhabdovirus oryzae; Rhabdoviridae*) has a single‐stranded RNA genome encoding a minimum of seven canonical proteins.^[^ ^3^ ^]^ RSMV‐infected plants typically display slight dwarfing and have twisted leaves with yellow stripes. These diverse rice RNA viruses cause plant dwarfing, necrotic stripes on newly developed leaves, or leaf wilting and dwarfism all resulting in serious yield losses.

Many studies show that different viruses employ diverse pathogenicity strategies for successful infection during the arms race between plant hosts and viruses.^[^ ^2^, ^4^, ^5^, ^6^ ^]^ However, there has been less study of common pathogenic mechanisms among diverse plant viruses. Recently, our studies showed that three independently evolved virus proteins with little sequence similarity (SRBSDV SP8, RSV P2, and RSMV M proteins) all regulate the essential components of plant hormone pathways to benefit viral infection and vector feeding. For example, they all have transcriptional repressor activity and are associate with the key components of jasmonic acid (JA) signaling, including JASMONATE ZIM DOMAIN (JAZ) repressor proteins, and transcription factors OsMYC2/3. These viral transcriptional repressors can directly disrupt the OsMED25‐OsMYC2/3 complex, inhibit the transcriptional activation of OsMYC2/3, and then preferentially combine with OsJAZ proteins to synergistically enhance their repression activity. In addition, these proteins manipulate the JA signaling pathway to overcome host defense to sucking vectors, thereby facilitating viral infection.^[^ ^7^ ^]^ These very different viruses therefore have a common strategy to hijack and repress the JA pathway in favor of both viral pathogenicity and vector transmission. Hence, understanding the general pathogenic mechanism adopted by these viruses is not only important for basic plant virology but may also be useful for developing broad‐spectrum antiviral strategies to prevent and control virus diseases.

For their survival, plants have complex defense strategies against viruses, including RNA silencing, R gene‐mediated resistance, and the activation of the phytohormone signaling network.^[^ ^5^, ^8^, ^9^ ^]^ Recent reports showed that plant hormones, especially JA, play vital roles in rice broad‐spectrum antiviral immunity against different rice viruses.^[^ ^7^, ^10^, ^11^ ^]^ JA, an oxygenated lipid‐based hormone, is a key regulator of plant development and stress responses to necrotrophic pathogens and insect infestation.^[^ ^12^, ^13^ ^]^ The JA signaling transduction pathway depends on the basic helix‐loop helix (bHLH) transcription factor family MYC2/3/4 transcription factors, of which MYC2 serves as a master regulator.^[^ ^14^ ^]^ In the absence of JA, JAZ proteins interact with MYCs and suppress the activation of these transcription factors. At the same time, JAZ proteins physically interact with the NINJA or EAR motif to recruit TOPLESS (TPL) to further suppress the activation of MYC transcription factors.^[^ ^15^, ^16^ ^]^ Upon JA perception, JA receptor coronatine insensitive 1 (COI1) specifically associates with JAZ proteins leading to ubiquitination and degradation, thus releasing JA‐responsive transcription factors to induce the expression of JA‐responsive genes. Many regulators were reported to participate in the JA signaling pathway in *Arabidopsis thaliana*.^[^ ^17^, ^18^, ^19^ ^]^ For instance, the histone acetyltransferase GCN5 can mediate Groucho/Tup1‐like corepressor TOPLESS (TPL) acetylation and increase its interaction with NINJA adaptor leading to transcriptional repression of MYC2 activity.^[^ ^20^ ^]^ In addition, the JAZ proteins can associate with the WD‐Repeat/bHLH/MYB complexes to suppress JA‐regulated stamen development.^[^ ^21^ ^]^ However, there are few regulators known to directly manipulate the JAZ‐MYC complex in rice and the relationships between the regulators involved in the JA pathway during the process of pathogen infection are not well understood.

In this study, we found that a plant‐specific RAV‐type transcription factor, OsRAV15, confers rice broad‐spectrum antiviral resistance by modulating the JA pathway. OsRAV15 physically interacts with the OsMYC2‐JAZ complex and functions cooperatively with OsMYC2 in JA signaling. Furthermore, different viral proteins directly interfered with OsRAV15‐mediated JA antiviral immunity to facilitate viral pathogenicity. Collectively, we conclude that the OsRAV15‐OsMYC2 module plays an essential role in fine‐tuning JA signaling, which improves our understanding of JA‐mediated antiviral immunity.

## 2 Results

### 2.1 Evolutionary‐Distinct Viral Proteins Interact with a RAV‐Type Transcription Factor OsRAV15

**Figure 1:**
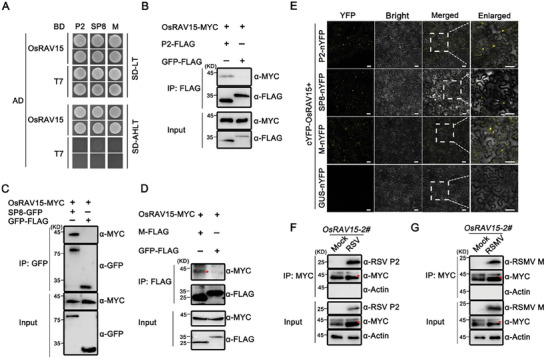
Distinct viral proteins interacted with OsRAV15. A) Y2H assays showing the interaction between OsRAV15 and different viral proteins (RSV P2, SRBSDV SP8, and RSMV M) in yeast cells. Viral proteins were cloned into pGBKT7 (BD), while OsRAV15 was cloned into the pGADT7 (AD) yeast vector. The different combinations were transformed into yeast cells and grown on SD‐L‐T plates at 30 °C for 3 days. Colony growth was scanned after 3 days of incubation in SD‐L‐T‐H‐Ade medium. B–D) Co‐immunoprecipitation (Co‐IP) assays showing that OsRAV15 interacted with viral proteins P2 (B), SP8 (C), and M (D) in vivo. OsRAV15‐MYC and P2‐FLAG, SP8‐GFP, M‐FLAG, or GFP‐FLAG (negative control) were transiently co‐expressed in *N. benthamiana* leaves. Total proteins were extracted, and the supernatants were precipitated with FLAG or GFP beads, followed by Co‐IP. The immunoprecipitated (IP) and input proteins were then analyzed using anti‐MYC and anti‐GFP or anti‐FLAG antibodies. The red asterisks represent the specific band. E) BiFC assays confirming the interactions of OsRAV15 with viral proteins P2, SP8, and M. cYFP‐OsRAV15 was co‐expressed with P2‐nYFP, SP8‐nYFP, M‐nYFP or the negative control Gus‐nYFP into *N. benthamiana* leaves. The white dotted box is an enlarged area. The images were captured by confocal microscopy at 48 hpi. Scale bar = 50 µm. F) Co‐IP assays of proteins isolated from healthy *OsRAV15* and RSV‐infected *OsRAV15* plants by RSV P2 and MYC antibody. The red asterisks represent the specific band. G) Co‐IP assays of proteins isolated from healthy OsRAV15 and RSMV‐infected *OsRAV15* plants by RSMV M and MYC antibody. The red asterisks represent the specific band. The precipitated proteins were analyzed by western blots with the indicated antibodies. Each experiment was repeated three times with similar results.

Our recent studies found that several unrelated rice viruses commonly repressed phytohormone‐mediated antiviral immunity by encoding a class of evolutionary‐distinct viral proteins (RSV P2, SRBSDV SP8, and RSMV M).^[^ ^7^ ^]^ To investigate the basis of this common mechanism, yeast two‐hybrid (Y2H) assays were employed to screen a rice cDNA library using the three different viral proteins as baits. A clone that encodes a RAV‐type transcription factor, OsRAV15 (LOC_Os02g25820), was found to interact with each of the viral proteins. We then cloned the full‐length of OsRAV15 and its homologous and found that only OsRAV15 specifically interacted with all three viral proteins in yeast cells (**Figure** **1**A; Figure S1A, Supporting Information), and Co‐IP assays demonstrated interactions *in planta* between these viral proteins and OsRAV15 protein (Figure 1B–D). Further BiFC assays gave consistent results. When different combinations of P2‐nYFP/SP8‐nYFP/M‐nYFP were co‐expressed with cYFP‐OsRAV15, there were strong YFP fluorescence signals in *N. benthamiana* leaves, while no fluorescence signal was observed in the combinations of cYFP‐OsRAV15/GUS‐nYFP (Figure 1E). The localization of the viral protein and OsRAV15 was mainly in the nucleus (Figure S1B, Supporting Information). We also confirmed these interactions in rice plants infected by these viruses (Figure 1F,G). Collectively, these results demonstrate that OsRAV15 is a common target of distinct viral proteins.

### 2.2 OsRAV15 Exhibits Broad‐Spectrum Antiviral Resistance

**Figure 2:**
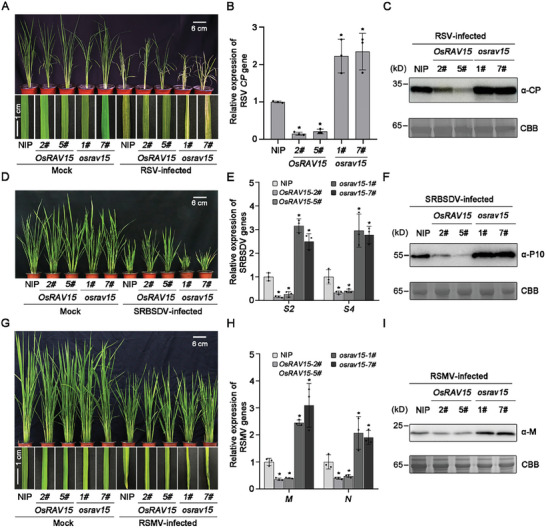
OsRAV15 confers resistance to different viral infections in rice. A) Symptoms of RSV infection in *OsRAV15* transgenic (*n* = 30) and mutant plants compared with NIP plants (*n* = 29). The areas of typical yellow stripes and death of the young leaves indicate the degree of disease symptoms. The phenotypes were observed and photos taken at 30 days post‐inoculation (dpi). Scale bars = 6 or 1 cm. B) RT‐qPCR results showing the relative expression levels of the RSV *CP* gene in RSV‐infected *OsRAV15*‐overexpressing transgenic, mutant, and NIP plants at 30 dpi. Error bars represent SD, values are means ± SD (*n* = 3 biologically independent replicates per genotype). Significant differences were analyzed using ANOVA followed by Tukey's multiple comparisons test. ^*^ at the columns indicate significant differences (*p* ≤ 0.05). C) The accumulation of RSV CP protein in RSV‐infected *OsRAV15*‐overexpressing transgenic, mutant, and NIP plants by western blotting. CBB serves as the loading control to monitor input protein amounts. D) Symptoms of SRBSDV infection in *OsRAV15*‐overexpressing transgenic (*n* = 24), mutant plants (*n* = 24) compared with NIP (*n* = 25) plants. The phenotypes were observed and photos taken at 30 dpi. Scale bars = 6 cm. E) RT‐qPCR results showing the relative expression levels of SRBSDV RNA segments (*S2* and *S4*) in SRBSDV‐infected *OsRAV15*‐overexpressing transgenic, mutant, and NIP plants at 30 dpi. Error bars represent SD, values are means ± SD (*n* = 3 biologically independent replicates per genotype). Significant differences were analyzed using ANOVA followed by Tukey's multiple comparisons test. ^*^ at the columns indicate significant differences (*p* ≤ 0.05). F) The accumulation of SRBSDV P10 protein in SRBSDV‐infected *OsRAV15*‐overexpressing transgenic, mutant, and NIP plants by western blotting. CBB serves as the loading control to monitor input protein amounts. G) Symptoms of RSMV infection in *OsRAV15*‐overexpressing transgenic (*n* = 25), mutant plants (*n* = 25) compared with NIP (*n* = 24) plants. The areas of yellow stripes on leaves followed by mosaic and occasional twisting of some leaves represent the degree of disease symptoms. The phenotypes were observed and photos were taken at 45 dpi. Scale bars = 6 or 1 cm. H) RT‐qPCR results showing the relative expression levels of RSMV *M* and *N* genes in RSMV‐infected *OsRAV15* transgenic, mutant, and NIP rice plants at 45 dpi. Error bars represent SD, values are means ± SD (*n* = 3 biologically independent replicates per genotype). Significant differences were analyzed using one‐way ANOVA followed by Tukey's multiple comparisons test. ^*^ at the columns indicate significant differences (*p* ≤ 0.05). I) The accumulation of RSMV M protein in RSMV‐infected *OsRAV15* transgenic, mutant, and NIP plants by western blotting. CBB serves as the loading control to monitor input protein amounts. And *p* values of statistic tests (B, E, H) were provided in Table S3 (Supporting Information).

Since three distinct viral proteins interacted with OsRAV15, we next investigated the role of OsRAV15 in rice antiviral defense. Firstly, we generated *OsRAV15* overexpressing transgenic plants (lines *OsRAV15‐2#* and *OsRAV15‐5#*) in a wild‐type *Nipponbare* (NIP) background. The expression levels of *OsRAV15* were verified in the T3 generation transgenic plants (Figure S2A,B, Supporting Information). The phenotypic characteristics of *OsRAV15* overexpressing transgenic were observed in the filling period. *OsRAV15* overexpressing plants had slightly lower plant height and delayed heading, but there was no difference in the number of tillers (Figure S3A–C, Supporting Information). After challenging with RSV, NIP plants exhibited discontinuous yellow stripes and necrosis, but symptoms on the transgenic lines overexpressing *OsRAV15* were milder than in the controls (**Figure** **2**A). Consistently, real‐time quantitative PCR (RT‐qPCR) and western blotting results showed that the transcription levels of RNA3 and protein levels of RSV coat protein (CP) were significantly reduced in transgenic plants overexpressing *OsRAV15* compared to control NIP plants (Figure 2B,C). Thus, these results show that rice overexpressing *OsRAV15* was more resistant to RSV infection.

To further substantiate the role of OsRAV15 in rice antiviral defense, CRISPR/Cas9 system‐based OsRAV15 mutants were generated in the background of NIP plants.^[^ ^22^ ^]^ We identified and isolated two homozygous mutants named *osrav15‐1#* and *osrav15‐7#* (Figure S4A, Supporting Information). Mutants had residues either deleted or inserted, resulting in frame‐shift or premature termination of the ORF (Figure S4B, Supporting Information). The phenotypic characteristics of mutant plants did not differ significantly from those of the control plants (Figure S3A–C, Supporting Information). After inoculation with RSV, the mutant plants had more severe symptoms (more severe curling or death of the young leaves) (Figure 2A). The RNA and protein of RSV CP accumulated to distinctly higher levels in *osrav15* lines than in NIP plants (Figure 2B,C). Together, these results confirm that OsRAV15 plays a positive role in controlling rice antiviral immunity to RSV.

In similar experiments with the other two viruses, transgenic plants overexpressing OsRAV15 were also more resistant to SRBSDV. Transgenic plants had milder dwarfing symptoms than the controls (Figure 2D) and reduced SRBSDV accumulation of RNAs (*S2* and *S4*) and SRBSDV P10 protein levels (Figure 2E,F). The *osrav15* mutant plants were more sensitive to SRBSDV infection than the controls, mainly reflected in more severe dwarfing symptoms (Figure 2D) and greater viral accumulation (Figure 2E,F). Likewise, in response to RSMV inoculation, transgenic plants overexpressing OsRAV15 had milder symptoms (increased tiller number, yellow streaks on the leaves, twisted or crinkled mosaic) than the controls whereas symptoms were more severe in *osrav15* mutants than in NIP controls (Figure 2G). Compared to the controls, the levels of RSMV *M* and *N* genes were lower in *OsRAV15* transgenic plants and higher in *osrav15* mutants (Figure 2H), and similar results were obtained by testing the protein levels using anti‐M antibody (Figure 2I). The transcriptional level of *the OsRAV15* gene was significantly induced after different viral infections (Figure S5, Supporting Information). Collectively, these results show that OsRAV15 exhibits a broad‐spectrum antiviral resistance against the *tenuivirus* RSV, the *fijivirus* SRBSDV and the *cytorhabdovirus* RSMV.

### 2.3 OsRAV15 Positively Regulates the JA Pathway

To further investigate the antiviral immunity mediated by OsRAV15, we conducted RNA sequencing (RNA‐seq) analysis of transgenic plants overexpressing *OsRAV15, osrav15* mutant with those in WT plants in response to SRBSDV infection. Differentially expressed genes (DEGs) were selected based on significance when log_2_(fold change) ≥ 1, *p* ≤ 0.05.^[^ ^23^ ^]^ Volcano plot analysis of all DEGs found that the expression of a large portion of responsive genes was modulated during the response to viral infection (Figure S6, Supporting Information). A Gene Ontology (GO) enrichment analysis of the DEGs showed that SRBSDV infection rapidly induced the expression of a large number of defense response genes, particularly those associated with the GO term “response to jasmonic acid” in *OsRAV15* overexpressing plants, while expression of these genes remained unchanged in *osrav15* mutants (Figure S6, Supporting Information). We analyzed the JA‐Ile content in *OsRAV15*‐overexpressing and *osrav15* mutant plants. The results revealed that the JA‐Ile content in *osrav15* mutant plants was significantly reduced compared with that in the control NIP plants, whereas *OsRAV15*‐overexpressing plants exhibited increased JA‐Ile levels (Figure S7, Supporting Information). Additionally, we also detected the expression analysis of classical JA pathway marker genes (e.g., *OsLOX2*, *OsAOS2*, and *OsJAZ9*) in OsRAV15‐related rice plants (Figure S8, Supporting Information). The expression patterns of these genes were consistent with the observed JA‐Ile levels, further supporting the role of OsRAV15 in regulating JA signaling.

**Figure 3:**
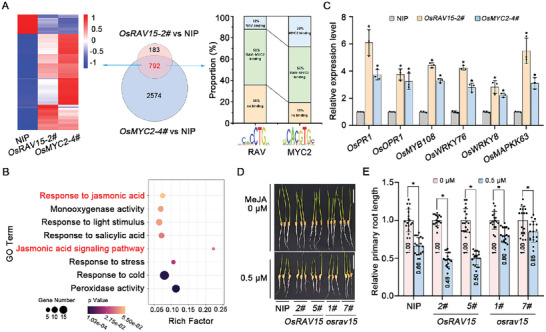
OsRAV15 positively regulates the JA pathway. A) Venn diagrams showing overlaps of differentially expressed genes (DEGs) in *OsRAV15‐2#* versus NIP and *OsMYC2‐4#* versus NIP samples (middle panel). The heat maps display hierarchical cluster analysis of the 792 (genes labeled in red) overlapping DEGs in the two comparison groups of *OsRAV15‐2#* versus NIP and *OsMYC2‐4#* versus NIP (left panel). The “12% RAV binding” refers to genes that exclusively have RAV binding sites, meaning genes that contain both RAV and MYC2 binding sites are excluded from this category. Similarly, the “29% MYC2 binding” represents genes with only MYC2 binding sites, excluding those that also contain RAV binding sites. The “52% shared binding” indicates the percentage of genes that have both RAV and MYC2 binding sites, reflecting overlap in their binding targets (right panel). The relative profile score threshold was set to 90%. All DEGs were selected using cutoff *p* ≤ 0.05 and fold‐change >2 compared with controls. B) Gene ontology (GO) enrichment analysis of 792 DEGs (labeled in red) in (A). “Rich factor” shows the ratio between the number of DEGs and the total genes in this pathway. C) RT‐qPCR analyses of *OsPR1, OsOPR1, OsMYB108, OsWRKY76, OsWRKY8* and *OsMAPKK63* genes expression levels in *OsRAV15*‐overexpressing and *OsMYC2*‐overexpressing plants. Error bars represent SD, values are means ± SD (*n* = 3 biologically independent replicates per genotype). Significant differences were analyzed using ANOVA followed by Tukey's multiple comparisons test. ^*^ at the columns indicate significant differences (*p* ≤ 0.05). D) Phenotypes of NIP, *OsRAV15*‐overexpressing, and mutant seedlings treated with 0.5 µm MeJA. At least 15 (*n* = 18) germinated seeds were placed in a culture solution containing different concentrations of MeJA for ≈5 days, scale bar = 2 cm. E) The primary root lengths of NIP, OsRAV15‐overexpressing, and mutant plants relative to the control plants. Values were obtained from at least 15 seedlings (*n* = 18). ^*^ at the top of the columns indicate significant differences (*p* ≤ 0.05).

To confirm whether OsRAV15‐mediated antiviral defense was associated with the JA signaling pathway, we performed RNA sequencing analysis to compare WT plants with those overexpressing either OsRAV15 or OsMYC2 (a master transcription factor in JA signaling) (**Figure** **3**A). A Venn diagram analysis indicated that 792 overlapping DEGs in the two comparison groups of *OsRAV15‐2#* versus NIP and *OsMYC2‐4#* versus NIP (Figure 3A, middle panel), suggesting that these genes were co‐regulated by OsRAV15 and OsMYC2. Further analysis showed that these overlapping DEG exhibited consistent expression patterns in *OsRAV15‐2#* and *OsMYC2‐4#* compared to NIP plants (Figure 3A, left panel). We further analyze the binding motifs of OsMYC2 and OsRAV15 in these 792 gene promoter regions. RAV‐type transcription factor was showed to bind the B3 motif (5′‐CACCTG‐3′),^[^ ^24^ ^]^ and MYC2 transcription factor binds to the G‐box motif (5′‐CACGTG/CACATG‐3′) in the promoters of their target genes.^[^ ^25^ ^]^ Promoter prediction analysis found that ≈12% of the genes contained the RAV15 binding site, and 29% contained the MYC2 binding site individually. Interestingly, more than half of the genes (≈52%) were found to be co‐regulated by OsRAV15 and OsMYC2 (Figure 3A, right panel). GO enrichment analysis showed that the overlapping DEGs were highly enriched in transcriptional regulation and various hormone stress responses. Interestingly, the terms “response to jasmonic acid” and “jasmonic acid mediated signaling pathway” were also enriched (Figure 3B). These results indicated that most of the genes regulated by OsRAV15 were also involved in OsMYC2‐mediated JA signaling. To validate the transcriptome data, the expression levels of several defense‐related genes were selected for RT‐qPCR analysis. The results showed that the expression levels of these defense genes (*OsPR1, OsOPR1, OsMYB108, OsWRKYs*, and *OsMAPKK63*) were significantly increased both in plants overexpressing *OsRAV15* and in those overexpressing *OsMYC2* compared with NIP (Figure 3C).

To assess the genes co‐regulated by both OsRAV15 and OsMYC2 in response to viral infection, we employed volcano plot analysis to examine the expression levels of the 792 DEGs regulated by both OsRAV15 and OsMYC2 in NIP plants upon SRBSDV infection. The results showed that a significant proportion of these genes, including 451 up‐regulated and 243 down‐regulated genes, exhibited alterations in response to viral infection in NIP plants (Figure S9A, Supporting Information). The RNA‐seq data were further validated by RT‐qPCR assay (Figure S9B, Supporting Information). These findings further demonstrate that OsRAV15 and OsMYC2 can co‐regulate a multitude of genes during viral infection. To further study the biological significance of OsRAV15 in JA signaling, we used the OsRAV15 transgenic plants to analyze JA sensitivity. As previously reported, JA treatment suppresses root growth in rice, and the inhibitory effect is enhanced when JA signaling is activated.^[^ ^7^ ^]^ We treated the seedling roots of *OsRAV15*‐overexpressing and *osrav15* mutant plants with 0.5 µm MeJA for 5 days in the dark. The root growth of control NIP plants was distinctly inhibited by MeJA treatment (Figure 3D,E) and this suppressive effect was clearly enhanced in plants overexpressing *OsRAV15* but significantly decreased in *osrav15* mutants (Figure 3D,E), indicating that OsRAV15 plays a positive role in activate JA signaling. Together, these data indicate that OsRAV15 participates in OsMYC2‐mediated JA signaling.

### 2.4 OsRAV15 Functions Synergistically with OsMYC2 in JA Signaling

**Figure 4:**
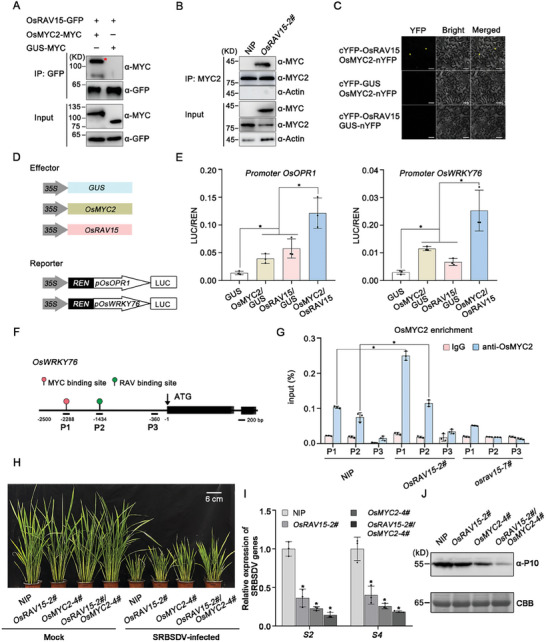
OsRAV15 is associated with the OsMYC2 transcription factor and co‐activated the JA signaling. A) Co‐IP assays indicating that OsRAV15 interacted with OsMYC2 in *N. benthamiana* leaves. OsRAV15‐GFP and OsMYC2‐MYC or GUS‐MYC (negative control) were transiently co‐expressed in *N. benthamiana* leaves. Total proteins were extracted, and the supernatant precipitated with GFP beads, followed by Co‐IP. The immunoprecipitated (IP) and input proteins were analyzed using anti‐GFP and anti‐MYC antibodies. The red asterisks represent the specific band. B) Co‐IP assays showing that OsRAV15 interacted with OsMYC2 in *OsRAV15‐2#* transgenic plants. Total proteins were extracted, the supernatant precipitated with Protein A/G OsMYC2 antibody beads, and the immunoprecipitated (IP) and input proteins were then analyzed using anti‐OsMYC2 and anti‐MYC antibodies. C) BiFC assays showing the interactions between OsRAV15 and OsMYC2 in *N. benthamiana* leaves. cYFP‐OsRAV15 co‐expressed with OsMYC2‐nYFP or the negative controls were injected into *N. benthamiana* leaves. The images were captured by confocal microscopy at 48 h post‐inoculation (hpi). Scale bar = 50 µm. D) Schematic diagram of the dual‐LUC assays. The promoters of *OsOPR1* and *OsWRKY76* with firefly luciferase (LUC) were used to construct the *pOsOPR1::LUC* and *pOsWRKY76::LUC* vectors as the reporters. Renilla luciferase (REN) was the internal control. OsMYC2, OsRAV15, and GUS (negative control) were the effectors. E) The dual‐LUC assays show that OsMYC2 collaborated synergistically with OsRAV15 to promote the transcription of *OsOPR1* and *OsWRKY76* in *N. benthamiana* leaves. The same concentration combinations of OsMYC2/GUS, OsRAV15/GUS, and OsMYC2/OsRAV15 were transiently expressed in *N. benthamiana* leaves. GUS was used as a negative control. The LUC/REN ratio represents the relative LUC activity. Error bars represent SD, values are means ± SD (*n* = 3 biologically independent replicates per genotype). Significant differences were analyzed using one‐way ANOVA followed by Tukey's multiple comparisons test. ^*^ at the columns indicate significant differences (*p* ≤ 0.05). F) Schematic diagram of *OsWRKY76* genes with exons displayed as black boxes for ChIP‐qPCR analyses. P1 contained MYC binding site; P2 contained RAV binding site. The number represents the specific location of P1, P2, and P3. The distance between P1 (including MYC binding site) and P2 (including RAV binding site) binding sites was ≈860 bp. The distance between P2 and P3 (no binding site) was ≈1074 bp. G) ChIP‐qPCR analyses of the enrichment of OsMYC2 in the *OsWRKY76* promoter using OsMYC2‐specific antibodies. 7 day‐old NIP, *OsRAV15‐2#* and *osrav15‐7#* seedlings were used in ChIP assays. Error bars represent SD, values are means ± SD (*n* = 3 biologically independent replicates per genotype). Significant differences were analyzed using ANOVA followed by Tukey's multiple comparisons test. ^*^ at the columns indicate significant differences (*p* ≤ 0.05). H) Viral symptoms in *OsRAV15‐2#* (*n* = 26), *OsMYC2‐4#* (*n* = 30), *OsRAV15‐2#/OsMYC2‐4#* (*n* = 28) transgenic plants and NIP (*n* = 30) in response to SRBSDV infection. The phenotypes were observed and photos taken at 40 dpi. Scale bars = 6 cm. I) The relative expression levels of SRBSDV RNA segments (*S2* and *S4*) in SRBSDV‐infected *OsRAV15‐2#, OsMYC2‐4#, OsRAV15‐2#/OsMYC2‐4#* transgenic plants and NIP rice plants as detected by RT‐qPCR at 40 dpi. Error bars represent SD, values are means ± SD (*n* = 3 biologically independent replicates per genotype). Significant differences were analyzed using ANOVA followed by Tukey's multiple comparisons test. ^*^ at the columns indicate significant differences (*p* ≤ 0.05). J) The accumulation of SRBSDV P10 protein in SRBSDV‐infected *OsRAV15‐2#, OsMYC2‐4#, OsRAV15‐2#/OsMYC2‐4#*, and NIP plants by western blotting. CBB serves as the loading control to monitor input protein amounts. And *p* values of statistic tests (I) were provided in Table S3 (Supporting Information).

Given that most of the genes regulated by the transcription factors OsRAV15 were involved in OsMYC2‐mediated JA signaling, we speculated that OsRAV15 might directly associate with OsMYC2 *in planta*. To confirm this hypothesis, Co‐IP assays were performed and the results showed that OsRAV15 specifically interacted with OsMYC2 in *N. benthamiana* (**Figure** **4**A) and rice transgenic plants (Figure 4B). To further verify the interactions between OsRAV15 and OsMYC2 transcription factors in plant cells, we conducted BiFC assays and the results showed interactions between OsRAV15 and OsMYC2 in the nucleus of plant cells (Figure 4C). In addition, IP‐MS experiments from NIP and *OsRAV15* overexpressing plants also display a clear interaction between OsRAV15 and OsMYC2 in rice (Table S1, Supporting Information). Our recent studies have shown that OsMYC3 also played an important role in the JA pathway,^[^ ^28^ ^]^ we then investigate the association of OsRAV15 with OsMYC3. The results showed that OsRAV15 was directly associated with OsMYC3 in plant cells by using CoIP and BiFC assays (Figure S10, Supporting Information). Based on the direct physical association of OsRAV15‐OsMYC in rice, we wondered whether OsRAV15 and OsMYC2 synergistically regulated the JA response. Interestingly, we found that the promoters of *OsOPR1* and *OsWRKY76* were predicted to contain the binding sites of both RAV‐type and MYC2. Hence, we further determined whether OsRAV15 and OsMYC2 bind to the promoters of *OsOPR1* and *OsWRKY76* and activate their expression. First, we fused the promoters of *OsOPR1* and *OsWRKY76* with firefly luciferase (LUC) for use in a dual‐luciferase transcriptional activity assay (Figure 4D). LUC expression driven by *OsOPR1* and *OsWRKY76* promoters were much higher when OsRAV15 and OsMYC2 were co‐expressed than when OsRAV15 or OsMYC2 were expressed alone, indicating that OsRAV15 and OsMYC2 not only bind to the *OsOPR1* and *OsWRKY76* promoters, but also synergistically activate gene expression (Figure 4E). Next, we conducted chromatin immunoprecipitation (ChIP) qPCR (ChIP‐qPCR) assays to analyze the enrichment of OsMYC2 and OsRAV15 at the *OsWRKY76* promoters using OsMYC2‐specific polyclonal antibodies in NIP, *OsRAV15‐2#* and *osrav15‐7#* plants. OsMYC2 is specifically bound to the G‐box motif in the promoters of the *OsWRKY76* gene. OsMYC2 polyclonal antibody was previously produced in our lab. The enrichment of the *OsWRKY76* promoter by OsMYC2 was significantly increased in *OsRAV15*‐overexpressing plants compared to NIP plants (Figure 4F,G). Additionally, the RAV binding site in the promoter of *OsWRKY76* was significantly enrichment in *OsRAV15*‐overexpressing plants than NIP plants, while the enrichment was obviously decreased in *osrav15* mutant plants (Figure 4G). These findings provide evidence supporting the co‐activation of gene expression by OsRAV15 and OsMYC2. Electrophoretic mobility shift assays (EMSA) showed that OsRAV15 or OsMYC2 all directly bound the promoters of *OsWRKY76*. Meanwhile, when both proteins were present, the binding signals to biotin‐labeled probes were significantly enhanced, indicating their synergistic interaction (Figure S11, Supporting Information). We next crossed *OsRAV15‐2#* with *OsMYC2‐4#* to generate *OsRAV15‐2#/OsMYC2‐4#* plants (Figures S2C,D and S3D–F, Supporting Information). The expression level of the *OsWRKY76* gene was significantly induced in *OsRAV15‐2#/OsMYC2‐4#* plants compared to *OsMYC2‐4#* and *OsRAV15‐2#* plants (Figure S12, Supporting Information). These results showed that OsRAV15 cooperates synergistically with OsMYC2 to activate the expression of JA‐related genes.

To clarify the relationship between OsRAV15 and OMYC2 in JA signaling, we tested the *OsRAV15‐2#/OsMYC2‐4#* hybrid plants phenotype in response to MeJA treatment. Consistent with the above results, *OsRAV15‐2#* plants exhibited increased sensitivity to MeJA treatment than control plants. Interestingly, the JA response of *OsRAV15‐2#/OsMYC2‐4#* plants was more sensitive than that of *OsRAV15‐2#* and *OsMYC2‐4#* plants individually (Figure S13, Supporting Information). To further investigate the role of OsRAV15‐OsMYC2 in JA‐mediated antiviral defense, we assessed the susceptibility of the crossed plants to SRBSDV infection. *OsRAV15‐2#/OsMYC2‐4#* hybrid plants demonstrated enhanced resistance to SRBSDV infection compared to NIP and overexpressing either *OsRAV15‐2#* or *OsMYC2‐4#* plants (Figure 4H–J). Similarly, hybrid plants were also more resistant to RSV infection (Figure S14, Supporting Information). Together, these results support the idea that OsRAV15 interacts with OsMYC2 to synergistically activate JA signaling.

### 2.5 OsJAZs Interact with OsRAV15 to Suppress the Synergistically Transcriptional Activation of OsRAV15‐OsMYC2 Module

**Figure 5:**
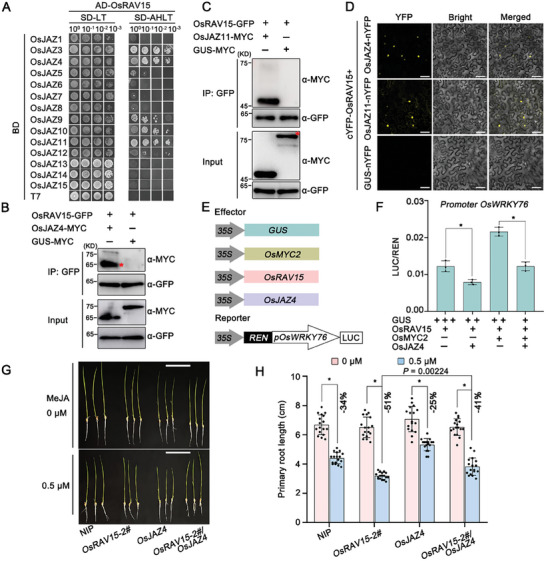
OsJAZs interact with OsRAV15 to suppress OsRAV15‐mediated transcriptional activity. A) OsRAV15 interacts with OsJAZs by Y2H assay. The 15 OsJAZ proteins except OsJAZ2 were cloned into pGBKT7 and tested for any interaction with OsRAV15. The different combinations were transformed into yeast cells and grown on SD‐L‐T plates at 30 °C for 3 days. Colony growth was scanned after 3 days of incubation in SD‐L‐T‐H‐Ade medium. B,C) Co‐IP assays indicating that OsRAV15 interacted with OsJAZ4 and OsJAZ11 in *N. benthamiana* leaves. OsRAV15‐GFP was transiently co‐expressed with OsJAZ4‐MYC, OsJAZ11‐MYC, or GUS‐MYC (negative control) in *N. benthamiana* leaves. Total proteins were extracted, and the supernatant precipitated with GFP beads, followed by Co‐IP. The immunoprecipitated (IP) and input proteins were analyzed using anti‐GFP and anti‐MYC antibodies. The red asterisks represent the specific band. D) BiFC assays confirm the interactions between OsRAV15 and OsJAZ4 or OsJAZ11 protein in *N. benthamiana* leaves. cYFP‐OsRAV15 was co‐expressed with OsJAZ4‐nYFP, OsJAZ11‐nYFP, or the negative controls into *N. benthamiana* leaves. The images were captured by confocal microscopy at 48 hpi. Scale bar = 50 µm. E) Schematic diagram of the dual‐LUC assays. The promoter of *OsWRKY76* with firefly LUC was used to construct the *pOsWRKY76::LUC* vector as the reporter. Renilla luciferase (REN) was the internal control. OsMYC2, OsRAV15, OsJAZ4, and GUS (negative control) were the effectors. F) The dual‐LUC assays indicating that OsJAZ4 not only interferes with the transcriptional function of OsRAV15 but also suppresses the synergistic effect of OsRAV15 and OsMYC2. The same concentration combinations of OsRAV15/GUS, OsRAV15/OsJAZ4/GUS, OsRAV15/OsMYC2/GUS, and OsRAV15/OsJAZ4/OsJAZ4/GUS were transiently expressed in *N. benthamiana* leaves. The LUC/REN ratio represents the relative LUC activity. Error bars represent SD, values are means ± SD (*n* = 3 biologically independent replicates per genotype). Significant differences were analyzed using one‐way ANOVA followed by Tukey's multiple comparisons test. ^*^ at the columns indicate significant differences (*p* ≤ 0.05). G) Phenotypes of NIP (*n* = 17), *OsRAV15* (*n* = 17), *OsJAZ4* (*n* = 17) and *OsRAV15/OsJAZ4* (*n* = 17) seedlings treated with 0.5 µm MeJA. The germinated seeds were placed in a culture solution containing different concentrations of MeJA for ≈5 days, scale bar = 2 cm. H) The primary root lengths of NIP, *OsRAV15, OsJAZ4*, and *OsRAV15/OsJAZ4* to the control plants. Values were obtained from at least 15 seedlings. ^*^ at the top of the columns indicate significant differences (*p* ≤ 0.05).

Previous work showed that JAZ proteins act as the essential negative components in JA signaling by repressing the transcriptional activation activity of MYC2. To investigate whether OsJAZ proteins directly regulate the transcriptional activation of OsRAV15, we first conducted Y2H assays and found that OsRAV15 interacted with multiple OsJAZ proteins (OsJAZ3, OsJAZ4, OsJAZ9, OsJAZ10, and OsJAZ11) in yeast cells (**Figure** **5**A). In Co‐IP experiments to further define the interaction between OsRAV15 and OsJAZ proteins, OsRAV15 had the strongest affinity for OsJAZ4 and OsJAZ11, while no bands were observed in GUS‐MYC combinations (Figure 5B,C). BiFC assays verified that a strong fluorescence was observed in the nucleus when cYFP‐OsRAV15 and OsJAZ4‐nYFP/OsJAZ11‐nYFP were co‐infiltrated but not in the negative control, indicating that OsRAV15 interacts with OsJAZ proteins in the nucleus (Figure 5D). Together, these results strongly support the conclusion that OsRAV15 associates with OsJAZ proteins *in planta*. Because OsJAZ4 interacted with both OsRAV15 and OsMYC2, we then tested whether OsJAZ4 directly influences the association of OsRAV15 and OsMYC2. The Co‐IP assays showed that the association between OsMYC2 and OsRAV15 was markedly decreased in the presence of OsJAZ4 protein (Figure S15, Supporting Information). Meanwhile, we further performed dual‐luciferase assays to investigate the influence of OsJAZ4 on the transcriptional activity of these two transcription factors. LUC expression driven by the *OsWRKY76* promoter was much lower when OsRAV15 was co‐expressed with OsJAZ4 than when OsRAV15 was expressed alone (Figure 5E,F; Figure S16A, Supporting Information). Meanwhile, the expression of LUC synergistically driven by both OsMYC2 and OsRAV15 was significantly suppressed in the presence of OsJAZ4 (Figure 5E,F). To further explore the biological significance of the suppression of the transcriptional activation activity of OsRAV15 by OsJAZ proteins, we conducted a JA sensitivity assay using transgenic plants overexpressing *OsRAV15, OsJAZ4*, and *OsRAV15/OsJAZ4* in comparison with NIP controls (Figure S2E,F, Supporting Information). We observed that *OsRAV15/OsJAZ4* (−41%) hybrid plants displayed decreased sensitivity to JA treatment compared to plants overexpressing *OsRAV15* alone (−51%) (Figure 5G,H). Together, these results showed that OsJAZ proteins directly interfere with the synergistic transcriptional activation of the OsRAV15‐OsMYC2 module.

### 2.6 Distinct Viral Proteins Suppress the Synergistic Transcriptional Activation of the OsRAV15‐OsMYC2 Module to Benefit Viral Infection

**Figure 6:**
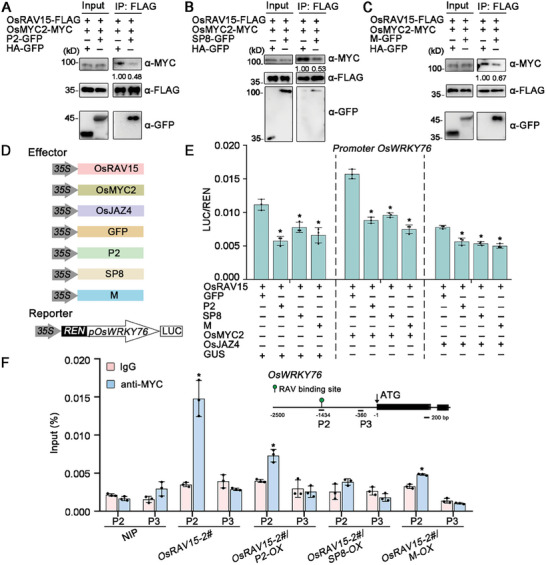
Viral proteins restrict OsRAV15 to activate JA antiviral signaling. A–C) Protein competition analyzed by Co‐IP assays. OsRAV15‐FLAG and OsMYC2‐MYC were infiltrated with or without P2‐GFP (A), SP8‐GFP (B), and M‐GFP (C) in leaves of *N. benthamiana*, HA‐GFP serves as a negative control. The samples were collected at 48 hpi for coimmunoprecipitation with FLAG beads. D) Schematic diagram of the dual‐LUC assays. The promoter of *OsWRKY76* with firefly LUC was used to construct the *pOsWRKY76::LUC* vector as the reporter. Renilla luciferase (REN) was the internal control. OsRAV15, OsMYC2, OsJAZ4, distinct viral proteins (P2, SP8, and M), and GFP (negative control) were the effectors. E) The dual‐LUC assays indicating that RSV P2, SRBSDV SP8, and RSMV M proteins all suppressed the cooperative function of OsRAV15 and OsMYC2 to activate the transcription of *the OsWRKY76* gene. To ensure the accuracy of the assay, the same concentration combinations were transiently expressed in *N. benthamiana* leaves. The LUC/REN ratio represents the relative LUC activity. Error bars represent SD, values are means ± SD (*n* = 3 biologically independent replicates per genotype). Significant differences were analyzed using one‐way ANOVA followed by Tukey's multiple comparisons test. ^*^ at the columns indicate significant differences (*p* ≤ 0.05). F) Schematic diagram of *OsWRKY76* genes with exons displayed as black boxes for ChIP‐qPCR analyses. P2 contained the RAV binding site. The number represents the specific location of P2 and P3. The distance between P2 (including RAV binding site) and P3 (no binding site) was ≈1074 bp. ChIP‐qPCR analyses of the enrichment of OsRAV15 in the *OsWRKY76* promoter using MYC‐specific antibodies. 30 d‐old NIP, *OsRAV15‐2#, OsRAV15‐2#/P2‐OX, OsRAV15‐2#/SP8‐OX* and *OsRAV15‐2#/M‐OX* seedlings were used in ChIP assays. Error bars represent SD, values are means ± SD (*n* = 3 biologically independent replicates per genotype). Significant differences were analyzed using one‐way ANOVA followed by Tukey's multiple comparisons test. ^*^ at the columns indicate significant differences (*p* ≤ 0.05).

Our recent studies have shown that distinct viral proteins (SRBSDV SP8, RSV P2, and RSMV M) all possess transcriptional repressor activity, and these viral proteins recruit OsJAZ proteins to cooperatively repress the transcriptional activity of OsMYC2.^[^ ^7^, ^26^, ^27^ ^]^ Since these viral proteins directly associate with both OsRAV15 and OsMYC2, we wondered whether they affect the association between OsRAV15 and OsMYC2. Competitive Co‐IP assays showed that the ability of OsRAV15 to associate with OsMYC2 was reduced in the presence of viral proteins in rice or *N. benthamiana* plants (**Figure** **6**A–C; Figures S17 and S18, Supporting Information). We also tested the effect of these viral proteins on the OsRAV15‐OsJAZs interaction. Competitive Co‐IP assays showed that these viral proteins have no obvious effect on the interaction intensity between OsRAV15 and OsJAZ4 (Figure S19, Supporting Information). This suggests that the distinct viral proteins all interfere with the interaction between OsRAV15 and OsMYC2 and that these viral proteins might inhibit the synergistic effect of OsRAV15 and OsMYC2 in the JA response.

We subsequently test whether these viral proteins impact the transcriptional activation ability of OsRAV15. The results revealed a significant reduction in the expression of *LUC* driven by the *OsWRKY76* promoter when OsRAV15 was co‐expressed with various viral proteins compared to co‐expression with the GFP control (Figure 6D,E; Figure S16B, Supporting Information). We also found the RSV P2, SRBSDV SP8, and RSMV M proteins all inhibited the cooperative function of OsRAV15 and OsMYC2 to activate the transcription of *OsWRKY76* gene (Figure 6E). Additionally, viral proteins and OsJAZ4 protein cooperatively repressed the transcriptional activation activity of OsRAV15 (Figure 6E). We next test whether viral proteins affect the binding of OsRAV15 to the promoter regions of *OsWRKY76* in vivo. We performed ChIP‐qPCR assays in *OsRAV15/P2‐OX, OsRAV15/SP8‐OX* and *OsRAV15/M‐OX* cross lines. The results showed that the ability of OsRAV15 bound to the promoter of *OsWRKY76* was significantly decreased in hybrid plants compared to *OsRAV15*‐overexpressing transgenic plant alone (Figure 6F). In addition, the expression level of the *OsWRKY76* gene was distinctly suppressed in *OsRAV15* and viral proteins hybrid transgenic plants compared to OsRAV15‐overexpression transgenic plants (Figure S20, Supporting Information). We hypothesize that viral proteins not only interfere with OsRAV15 binding to the OsWRKY76 promoter but may also suppress *OsWRKY76* expression by targeting other transcription factors that regulate this gene. These results showed that different viral proteins interfered with the binding of OsRAV15 to *OsWRKY76* promoter region. Together, these results suggest that distinct viral proteins associated with OsJAZ protein to cooperatively repress the OsRAV15‐OsMYC2 module‐mediated transcriptional activity.

**Figure 7:**
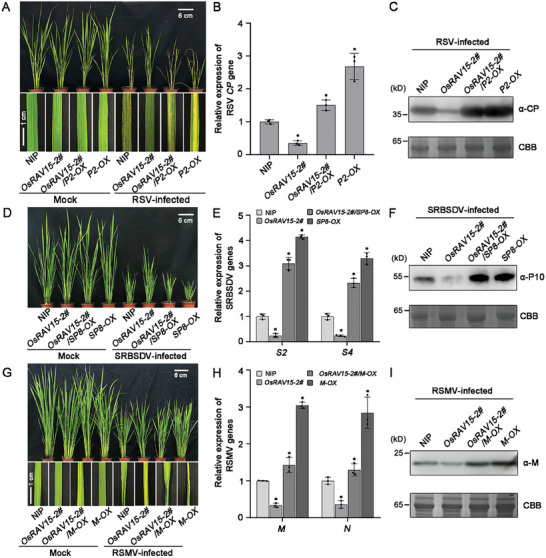
Distinct viral proteins impaired OsRAV15‐mediated resistance. A) Symptoms of RSV infection in *OsRAV15‐2#* (*n* = 28), *OsRAV15‐2#/P2‐OX* (*n* = 30) and *P2‐OX* (*n* = 30) plants compared with NIP (*n* = 28) plants. The areas of typical yellow stripes and death of the young leaves indicate the degree of disease symptoms. The phenotypes were observed and photos taken at 30 dpi. Scale bars = 6 or 1 cm. B) RT‐qPCR results showing the relative expression levels of the RSV *CP* gene in RSV‐infected *OsRAV15‐2#, OsRAV15‐2#/P2‐OX*, *P2‐OX*, and NIP plants at 30 dpi. Error bars represent SD, values are means ± SD (*n* = 3 biologically independent replicates per genotype). Significant differences were analyzed using one‐way ANOVA followed by Tukey's multiple comparisons test. ^*^ at the columns indicate significant differences (*p* ≤ 0.05). C) The accumulation of RSV CP protein in RSV‐infected *OsRAV15‐2#, OsRAV15‐2#/P2‐OX, P2‐OX*, and NIP rice plants by western blotting. CBB serves as the loading control to monitor input protein amounts. D) Symptoms of SRBSDV infection in *OsRAV15‐2#* (*n* = 25)*, OsRAV15‐2#/SP8‐OX* (*n* = 29) and *SP8‐OX* (*n* = 30) plants compared with NIP (*n* = 25) plants. The phenotypes were observed and photos taken at 30 dpi. Scale bars = 6 cm. E) RT‐qPCR results showing the relative expression levels of SRBSDV RNA segments (*S2* and *S4*) in SRBSDV‐infected *OsRAV15‐2#, OsRAV15‐2#/SP8‐OX, SP8‐OX* and NIP rice plants at 30 dpi. Error bars represent SD, values are means ± SD (*n* = 3 biologically independent replicates per genotype). Significant differences were analyzed using one‐way ANOVA followed by Tukey's multiple comparisons test. ^*^ at the columns indicate significant differences (*p* ≤ 0.05). F) The accumulation of SRBSDV P10 protein in SRBSDV‐infected *OsRAV15‐2#, OsRAV15‐2#/SP8‐OX, SP8‐OX*, and NIP plants by western blotting. CBB serves as the loading control to monitor input protein amounts. G) Symptoms of RSMV infection in *OsRAV15‐2#* (*n* = 30)*, OsRAV15‐2#/M‐OX* (*n* = 24) and *M‐OX* (*n* = 25) plants compared with NIP (*n* = 28) plants. The areas of yellow stripes on leaves followed by mosaic and occasional twisting of some leaves represent the degree of disease symptoms. The phenotypes were observed and photos were taken at 45 dpi. Scale bars = 6 or 1 cm. H) RT‐qPCR results showing the relative expression levels of RSMV *M* and *N* genes in RSMV‐infected *OsRAV15‐2#, OsRAV15‐2#/M‐OX, M‐OX*, and NIP rice plants at 45 dpi. Error bars represent SD, values are means ± SD (*n* = 3 biologically independent replicates per genotype). Significant differences were analyzed using one‐way ANOVA followed by Tukey's multiple comparisons test. ^*^ at the columns indicate significant differences (*p* ≤ 0.05). I) The accumulation of RSMV M protein in RSMV‐infected *OsRAV15‐2#, OsRAV15‐2#/M‐OX, M‐OX*, and NIP plants by western blotting. CBB serves as the loading control to monitor input protein amounts. And *p* values of statistic tests (B,E,H) were provided in Table S3 (Supporting Information).

To further investigate whether OsRAV15‐mediated resistance was impaired by these distinct viral proteins, we analyzed the function of OsRAV15 on virus infection in the presence of P2, SP8, or M. We first evaluated the effect of OsRAV15 on RSV infection in *OsRAV15/P2* hybrid plants. As observed previously, expression of RSV P2 protein resulted in more severe disease symptoms.^[^ ^27^ ^]^ Following inoculation with RSV, there were more severe yellow stripe symptoms in plants expressing P2 protein and OsRAV15 than in those expressing only OsRAV15 (**Figure** **7**A). The RNA and protein levels of RSV CP were also significantly higher in *OsRAV15‐2#/P2‐OX* lines than in *OsRAV15‐2#* plants (Figure 7B,C). In similar assays using SRBSDV, transgenic plants overexpressing OsRAV15 had milder dwarfing symptoms than control NIP plants, while *OsRAV15/SP8* hybrid plants were more sensitive to SRBSDV infection (similar differences to the control) (Figure 7D). RT‐qPCR and western blotting analysis showed that the levels of SRBSDV RNAs (*S2* and *S4*) and coat protein P10 in *OsRAV15‐2#/SP8‐OX* were much higher than in *OsRAV15‐2#* plants (Figure 7E,F). When inoculated with RSMV, the *OsRAV15‐2#* lines exhibited slight dwarfing, and yellow stripes on leaves, while expression of RSMV M protein in *OsRAV15‐2#* plants caused more severe twisting of some leaves (Figure 7G). The expression levels of RSMV RNAs (*M* and *N*) were also always higher in OsRAV15/M hybrid plants than in *OsRAV15‐2#* plants (Figure 7H), and similar results were observed in western blotting assays (Figure 7I). In addition, to further explore how these hybrid lines respond to JA treatment, we conducted JA sensitivity assays using transgenic plants overexpressing *OsRAV15, OsRAV15‐2#/P2‐OX, OsRAV15‐2#/SP8‐OX*, *OsRAV15‐2#/M‐OX* and NIP controls (Figure S21, Supporting Information). These results also suggested that viral proteins suppress OsRAV15 to activate JA signaling. Collectively, these results imply that OsRAV15‐mediated antiviral resistance is subverted by each of the different viral proteins P2, SP8, and M, thus benefiting the infection of their respective RNA viruses. In summary, we found that OsRAV15 is a transcriptional activator that cooperates with OsMYC2 to enhance JA signaling and is commonly targeted by independently evolved viral proteins (Figure S22, Supporting Information).

## 3 Discussion

Host‐virus interactions have complex regulatory networks. Plants have various sophisticated strategies to overcome the threat of pathogen infection, including the role of plant hormones as part of the host immune response to viral infection.^[^ ^5^, ^28^ ^]^ The crosstalk among various plant hormones plays a crucial role in the plant antiviral response. Different plant hormones, such as salicylic acid (SA), JA, auxins, abscisic acid (ABA), ethylene (ET), gibberellin (GA), and brassinosteroids (BRs), have distinct functions and their signaling networks play a key role in regulating plant‐virus interactions.^[^ ^5^, ^29^, ^30^ ^]^ As a counter‐defense, our previous research showed that various rice viruses have evolved viral transcriptional repressors that target crucial components of hormone pathways and inhibit their functions, thereby overcoming host resistance.^[^ ^7^, ^26^, ^27^, ^31^ ^]^ The independently evolved viral proteins (RSV P2, SRBSDV SP8, and RSMV M) interact with and impair the transcriptional complex to repress JA signaling.^[^ ^7^ ^]^ Beyond targeting JA signaling, these distinct viral proteins also interfered with the same key components of auxin, GA, and SA.^[^ ^26^, ^27^, ^31^, ^32^ ^]^ Thus, the manipulation of essential regulatory factors in plant hormones by rice viruses appears to be a significant and conserved anti‐defense strategy. In this study, we found that three different viral proteins, RSV P2, SRBSDV SP8 and RSMV M, interfere with the interaction between OsRAV15 and OsMYC2 (Figure 6), and associate with OsJAZ protein to co‐repress the OsRAV15‐OsMYC2 module‐mediated transcriptional functions (Figure 6D–F). We confirmed that OsRAV15‐mediated antiviral resistance was greatly subverted by P2, SP8, and M proteins (Figure 7). Our study unravels a common viral counter‐defense strategy in which different types of viruses subvert OsRAV15‐mediated broad‐spectrum resistance.

RAV transcription factors play key roles in many aspects of plant growth and development, as well as in plant responses to biotic and abiotic stresses.^[^ ^32^, ^33^, ^34^ ^]^ In *Arabidopsis*, inhibition of the expression of RAV1 and BES1 regulated plant growth and development.^[^ ^24^ ^]^ In addition to plant developmental processes, RAV transcription factors were also involved in the stress response of plants. The overexpression of the pepper *CARAV1* gene enhanced tolerance to the bacterial pathogen *Pseudomonas syringae* pv. tomato (*Pst*) DC3000 by inducing the expression of several pathogenesis‐related genes.^[^ ^35^ ^]^ *RAV2* (*SIRAV2*) transgenic tomato plants show resistance to *Ralstonia solanaceaerum* by activating the expression of *PR* genes.^[^ ^36^ ^]^ Therefore, these findings suggest that RAV transcription factors play important roles in the development and response to infection by different pathogens. While research has shown that the RAV family plays an important role in the process of plant resistance to pathogen infection, it is not fully understood how rice RAVs transcription factors work in rice‐pathogen interactions, especially those between rice and viruses. In this study, we found that overexpressing‐OsRAV15 increased rice resistance to different RNA viruses (*tenuivirus, fijivirus* and *cytorhabdovirus*), while mutant plants were more sensitive to viral infection (Figure 2). This suggested that OsRAV15 could provide broad‐spectrum antiviral resistance in monocotyledonous rice.

Previous studies have shown that RAV transcription factors can regulate the expression of genes involved in plant hormone signal transduction. For instance, RAV1 and RAV2 transcription factors can interact with ET signal transduction‐related genes, participating in the ethylene signal transduction process in *Arabidopsis*. Additionally, RAV1 and BES1 co‐regulated a large number of genes in brassinosteroids (BR) signaling processes to balance between growth and defense. However, the specific mechanism by which RAV transcription factors are involved in plant hormone signal transduction still needs further research and exploration. Here, we found that the JA signaling pathways were activated in transgenic plants overexpressing *OsRAV15* (Figure 3). To explore the OsRAV15‐mediated antiviral mechanism, we found that OsRAV15 is physically associated with the OsMYC2 transcription factor to synergistically activate the transcription of target genes (Figure 4A–G). The genes mentioned in our study, such as *OsWRKY76* and *OsOPR1*, are potential downstream targets of the OsMYC2 transcription factor. However, it is acknowledged that OsMYC2 likely regulates a multitude of downstream target genes that influence plant immunity, which requires us to be identified and characterized in the future. JAZ repressors regulate JA signaling by interacting with downstream transcription factors. We now show that OsJAZ4 protein suppresses the transcriptional activation of OsRAV15 in the JA signaling pathway (Figure 5E,F). These results show that OsRAV15 acts together with JAZ repressors to regulate JA signaling. Thus, we have identified a novel transcription factor, OsRAV15, involved in the JA pathway, providing the first evidence that JA signaling can be modulated by an OsRAV transcription factor to affect plant antiviral immunity.

## 4 Conclusion

In summary, we demonstrate that the independently evolved viral proteins (RSV P2, SRBSDV SP8, and RSMV M) target the same transcription factor OsRAV15 to repress its antiviral immunity response. Our earlier reports have shown that several different plant RNA viruses (RSV, SRBSDV, and RSMV) all target the host factors OsARF17, OsMYC2/3, and SLR1. From this perspective, manipulating key defensive hormonal pathways appears to be an important and conserved antiviral strategy to help viruses overcome plant defense barriers. However, the mechanism by which these different viral proteins target the same key host factor is still unclear. In the future, we will focus on analyzing structural similarities between these different viral proteins.

## 5 Experimental Section

### Plant Materials and Growth Conditions

Except where stated, all rice plants used in this study were cv *Niponbarre* (NIP) or generated in an NIP background. To generate transgenic plants overexpressing *OsRAV15, OsMYC2*, or *OsJAZ4*, the coding sequences of OsRAV15, OsMYC2, and OsJAZ4 were cloned individually into pCAMBIA1300 vector, driven by the CaMV 35S promoter, with downstream MYC, MYC or HA tag, respectively. These constructs were then transformed into *Agrobacterium tumefaciens* strain EHA105 for subsequent infection of rice callus. The *osrav15* knockout mutant lines were created using a CRISPR/Cas9‐based technology by BioRun (Wuhan, China). The hybrid rice plants *OsRAV15‐2#/OsMYC2‐4#, OsRAV15‐2#/OsJAZ4, OsRAV15‐2#/P2‐OX, OsRAV15‐2#/SP8‐OX* and *OsRAV15‐2#/M‐OX* were generated by genetic crosses using standard techniques. RSV‐infected plants were kindly provided by Professor Tong Zhou (Jiangsu Academy of Agricultural Sciences, China). SRBSDV‐ and RSMV‐infected plants were kindly provided by Professor Tong Zhang (South China Agricultural University, China). Rice plants were grown in an artificial growth chamber at 28–30 °C with a 14/10 h light/dark cycle. The *N. benthamiana* plants were grown in black plastic bowls at 22–25 °C and a 16/8 h photoperiod prior for two weeks.

### Insect Vectors and Viral Inoculation

RSV and SRBSDV were transmitted by the small brown planthopper (*Laodelphax striatellus*, SBPH) and white‐backed planthopper (*Sogatella furcifera*, WBPH), while RSMV was transmitted by the leafhopper (*Recilia dorsalis*). For viral inoculation, different viruses were inoculated by their insect vectors as described. Virus‐free nymphs of SBPH, WBPH, or leafhopper were fed on RSV‐, SRBSDV‐and RSMV‐infected plants for 4 days, and then removed onto 14‐day‐old healthy Wuyujing3 rice seedlings for 12 days to complete the circulation of the virus in vector insects. For virus transmission, SBPH carrying RSV, WBPH carrying SRBSDV, or leafhopper carrying RSMV were transferred to rice plants (about three viruliferous insects per seedling) at the 3 to 4‐leaf stage for 3 days. In the meantime, the rice seedlings were infested with the same number of virus‐free insects as a negative control. After 3 days of feeding, the insects were removed completely and plants were grown in the greenhouse to observe symptoms. For viral inoculation assays, each experiment used 20–30 seedlings. Infection of RSV, SRBSDV, or RSMV in these inoculated plants was confirmed by real‐time PCR (RT‐PCR) at 30 days post‐inoculation (dpi). The specific primers used to test for viral infection are listed in Table S2 (Supporting Information).

### Bimolecular Fluorescence Complementation (BiFC) Assays

For BiFC assays, the cDNA sequences encoding the OsRAV15, OsMYC2, OsMYC3, OsJAZ4, OsJAZ11, RSV P2, SRBSDV SP8, and RSMV M were amplified by PCR with and then individually inserted into the N‐terminus of YFP or the C‐terminus of YFP vectors. The recombinant plasmids were inserted into *Agrobacterium tumefaciens* strain GV3101 by electroporation. The different vectors used were as follows: cYFP‐OsRAV15, OsMYC2‐nYFP, OsMYC3‐nYFP, OsJAZ4‐nYFP, OsJAZ11‐nYFP, P2‐nYFP, SP8‐nYFP and M‐nYFP. The different vector combinations were infiltrated into *N. benthamiana* leaves for 48 h and the YFP fluorescence was detected by a confocal microscope (Leica TCS SP10). To capture the YFP signals, 514 nm excitation laser wavelength was used. All the primers used are listed in Table S2 (Supporting Information).

### Yeast Two‐Hybrid Assays (Y2H)

To analyze the interactions of OsRAV15 with JAZ proteins or viral proteins (RSV P2, SRBSDV SP8 and RSMV M), the full‐length coding sequences were inserted into pGBKT7 to produce the bait vectors (BD‐OsJAZs, BD‐P2, BD‐SP8 and BD‐M), whereas the full‐length OsRAV15 coding sequence was cloned into pGADT7 to generate the prey construct (AD‐OsRAV15). The recombinant plasmids with various interaction combinations were co‐transformed into the yeast strain AH109 (Clontech, CA, USA). The isolated colonies were grown on a dropout medium lacking Leu and Trp (SD/‐L‐T), and then the positive colonies were transferred onto SD/‐Leu/‐Trp/‐His/‐Ade selection plates for 3 days at 30 °C. The yeast growth was photographed for the interaction test. All the primers used are listed in Table S2 (Supporting Information).

### Co‐Immunoprecipitation (Co‐IP) Assays

For Co‐IP assays, the full‐length sequences of OsRAV15, OsMYC2, OsJAZ4, OsJAZ11, RSV P2, SRBSDV SP8, and RSMV M were amplified by PCR and then individually inserted into vector pCAMBIA1300, driven by the CaMV 35S promoter with MYC, FLAG, GFP and HA tags, respectively. The different combinations were co‐expressed in *N. benthamiana* leaves. For competitive Co‐IP assays, components to be detected were mixed in equal volumes before infiltration into *N. benthamiana*. For competitive Co‐IP assays in rice, the hybrid rice plants *OsRAV15‐2#/P2‐OX, OsRAV15‐2#/SP8‐OX*, and *OsRAV15‐2#/M‐OX* were used. The protein was extracted using extraction buffer (Thermo Scientific, Cat. no. 87788) containing 10 mm DTT, 1 × EDTA‐free protease inhibitor cocktail (Roche, Basel, Switzerland) for 30 min at 4° C. Immunoprecipitation assays were performed using Pierce anti‐c‐Myc magnetic beads (Thermo Scientific, USA), anti‐FLAG M2 beads (Sigma–Aldrich, USA), anti‐GFP‐trap beads (Chromotek, Germany) and Protein A/G OsMYC2 antibody beads, respectively for ≈2 h at 4° C (with gentle shaking). The agarose beads were washed three times with 1 × PBS. Then the co‐immunoprecipitated protein was detected by immunoblotting using anti‐FLAG (1:5000 dilution, Cat#HT201‐01, TransGen), anti‐MYC (1:5000 dilution, Cat#HT101‐01, TransGen), anti‐GFP (1:5000 dilution, Cat#HT801‐01, TransGen), anti‐HA (1:5000 dilution, Cat#HT301‐01, TransGen) and anti‐OsMYC2 (1:5000 dilution) antibodies. All the primers used are listed in Table S2 (Supporting Information).

### Dual Luciferase Transient Transcriptional Activity Assays (Dual‐LUC)

For luciferase assays, the promoter regions of *OsOPR1* and *OsWRKY76* were inserted into the pGreenII0800‐Luc vector (*pOPR1* and *pWRKY76*) as reporters. GUS‐MYC, OsRAV15‐MYC, OsMYC2‐MYC, OsJAZ4‐MYC, P2‐GFP, SP8‐GFP, and M‐GFP were used as effectors. The vectors of effectors and reporters were transformed into *Agrobacterium tumefaciens* strain GV3101. The different combinations with the same concentration were co‐expressed in *N. benthamiana* leaves for 48 h. Two discs were collected from the leaves using a puncher for shattering by an automatic oscillator. The dual‐LUC assays were performed using the Luciferase Reporter Assay System (Promega, Madison, Dual‐Luciferase Reporter Assay System #E1910). The LUC/REN ratio was used to quantify the promoter activity. At least three biological repeats were conducted for all experiments. All the primers used are listed in Table S2 (Supporting Information).

### Total RNA Extraction and RT‐qPCR

Total RNA was isolated from rice seedlings using TRIzol reagent (Invitrogen, Carlsbad, CA, USA). The cDNA was synthesized using the fast quant RT kit (Vazyme, Nanjing, China). Briefly, ≈1.0 µg DNase‐treated RNA was reverse transcribed in a 10 µL reaction volume. The resulting cDNA was used as the template for RT‐PCR and RT‐qPCR. RT‐qPCR was carried out on ten‐fold diluted cDNA by the ChamQTM SYBR qPCR Master Mix (Low ROX Premixed) and ABI7900HT Sequence Detection System (Applied Biosystems, Carlsbad, CA, USA). The relative expression levels of genes were normalized using the rice actin gene OsUBQ5 (AK061988). Data were analyzed by the 2^−ΔΔCt^ method and shown as means ± SD (*n* = 3). At least three biological repeats were conducted for all experiments. The RT‐qPCR primer sequences used are listed in Table S2 (Supporting Information).

### Chromatin Immunoprecipitation (ChIP)‐qPCR

The ChIP assays were performed according to the EpiQuikTM Plant ChIP Kit (Epigentek, Brooklyn, USA) following the manufacturer's instructions. Rice leaf tissue of 30‐days‐old NIP, *OsRAV15‐2#, osrav15‐7#, OsRAV15‐2#/P2‐OX, OsRAV15‐2#/SP8‐OX* and *OsRAV15‐2#/M‐OX* were harvested. The seedlings were cut into 2 mm strips and 1 g tissue was crosslinked with 1% formaldehyde by vacuum infiltration for 1 h at room temperature. 0.125 m Glycine was added to a final concentration of 100 mm to quench crosslinking under vacuum for 10 min. The samples were washed twice with ice‐cold double‐distilled water and tissue was frozen in liquid nitrogen. The chromatin was sheared into 200–1000 bp fragments by ultrasonic disruption. The fragmented chromatin solution was immunoprecipitated by ChIP grade anti‐MYC (Abcam) polyclonal antibody (1:2000 dilution) or anti‐OsMYC2 polyclonal antibody (1:2000 dilution, produced in the lab) bound Protein A/G‐Magnetic beads for 2 h. Negative control samples were prepared using immunoglobulin G (IgG). DNA fragments and input control were cleaned up following the manufacturer's instructions and then the DNA was used for qPCR using primers listed in Table S2 (Supporting Information). The relative enrichment was calculated as a percentage of the input. At least three biological repeats were conducted for all experiments.

### EMSA Assays

About 50‐bp sequences harboring the motifs were selected to generate the non‐labeled or biotin‐labeled probes. The probes were synthesized by sangon biotech (Table S2, Supporting Information). The full‐length of OsMYC2 and OsRAV15 were cloned individually into the pET32a vector and pGEX4T2 vector, respectively, to express HIS‐tagged or GST‐tagged fusion protein. The fusion protein was expressed in *E. coli* BL21(DE3) and was purified for EMSA assay. The EMSA assay was performed as described using the LightShift Chemiluminescent EMSA kit (Thermo Scientific) according to the manufacturer's protocols.

### JA Sensitivity Analysis

Inhibition of the rice primary root growth was used as an indicator of JA sensitivity. For JA sensitivity assays, the tested rice seeds were germinated and then transferred into rice nutrient solution containing 0.5 µm MeJA under short‐day conditions (8 h light/16 h dark, 30 °C for 5 days) for 5 days. The primary root lengths were measured and the phenotypes were photographed to record the sensitivity of the JA response. At least 15 transgenic seedlings were used for each line.

### Analysis JA‐Ile Concentration

Rice leaves of transgenic *OsRAV15‐2*#, *OSRAV15‐5#*, *osrav15‐1#*, *osrav15‐7#*, and NIP were collected at 30‐day‐old and powdered in liquid nitrogen, respectively. Samples (≈0.4 g of leaf powder) were extracted in 5 mL of 80% methanol solution (containing 0.1% formic acid) and purified by an Oasismode anion exchange (MAX) solid phase extraction (SPE) column. JA‐Ile was extracted and analyzed by ultra‐high‐performance liquid chromatography‐triple quadrupole mass spectrometry (UPLC‐MS/MS). The UPLC system included Nexera LC‐30AD UPLC System (Shimadzu) and an ACQUITY BEH C_18_ column (50 × 2.1 mm, 1.8 µm). For analysis of JA‐Ile using the ESl (Electron Spray lonization, Qtrap 6500 plus) source in negative ion mode, with 4.5 kV ion spray voltage, 60 declustering potential, and 26 and 20 collision energy respectively. Quantitative data were processed by the MultiQuant 3.0.3 software. The sample was replicated three times, each of which consisted of at least 4–5 pooled plants.

### RNA‐Seq and Analysis of Transcriptome Data

The *OsRAV15‐2#, OsMYC2‐4#*, and mock leaf samples of 30 days were collected (quick frozen with liquid nitrogen) and ground into powder. Total RNA examination, library construction, and sequencing were conducted by LC Bio (Hangzhou, China) by the Illumina Hiseq 2000/2500 platform. Mapping of sequencing reads to the rice genome (The MSU Rice Genome Annotation Project Database version 7.0) was done by Bowtie software. The genes were selected as DEGs based on significance when log_2_ fold change ≥ 1, *p* ≤ 0.05. Functional analysis of the DEGs was carried out using Gene Ontology (GO) (http://geneontology.org/) and Kyoto Encyclopedia of Genes and Genomes (http://www.genome.jp/kegg) pathways enrichment analyses tools. Three biological repeats and three to five leaves were collected from different seedlings for each biological repeat. Promoter prediction analysis of 792 overlapping differentially expressed genes. The 2500 bp upstream sequences of the transcription start site (TSS) were used to identify the RAV and MYC2 binding sites using the JASPAR database (http://jaspar2016.genereg.net/cgi‐bin/jaspar_db.pl?rm=browse&db=core&tax_group=plants). The relative profile score threshold was set to 90%.

### Multiple Sequence Alignment and Phylogenetic Analysis

The amino acid sequence encoding CDS of RAV proteins from rice was downloaded for phylogenetic analysis. ClustalW was used to align all acquired sequences. MEGA 6.0 software was used to construct a phylogenetic tree with 1000 bootstrap tests based on neighbor‐joining (NJ) methods.

### Statistical Analysis

Statistical significance analysis, quantitative real‐time PCR analysis, and dual‐luciferase reporter system were analyzed using one‐way ANOVA with Tukey's least significant difference tests. Each experiment was repeated at least three times, and data were represented as the mean. A *p* ≤ 0.05 was considered statistically significant, and asterisks indicate the statistical significance: ^*^, *p* ≤ 0.05. All analyses were performed using ORIGIN 8.0 software. For immunoblot quantification analysis, the intensities of bands were quantified with Image J.

### Accession Numbers

Sequence data from this article can be found in the rice genome annotation project database under the following accession numbers:

OsRAV15, Os02g25820; OsMYC2, Os10g42430; OsMYC3, Os01g50940; OsJAZ1, Os04g55920; OsJAZ3, Os08g33160; OsJAZ4, Os09g23660; OsJAZ5, Os04g32480; OsJAZ6, Os03g28940; OsJAZ7, Os07g42370; OsJAZ8, Os09g26780; OsJAZ9, Os03g08310; OsJAZ10, Os03g08330; OsJAZ11, Os03g08320; OsJAZ12, Os10g25290; OsJAZ13, Os10g25230; OsJAZ14, Os10g25250; OsJAZ15, Os03g27900; OsPR1, Os01g28450; OsOPR1, Os06g11290; OsMYB108, Os09g36730; OsWRKY76, Os09g25060; OsWRKY8, Os05g50610; OsMAPKKK63, Os01g50370, OsLOX2, Os08g39840; OsPR10, AF395880.

## Conflict of Interest

The authors declare no conflict of interest.

## Author Contributions

H.Z. and Z.S. designed the experiments. H.Z., C.H., C.G., and W.S. performed the experiments; H.Z., W.Y., X.H., L.L., Z.W., Y.L., and Z.S. analyzed the data; H.Z. and Z.S. wrote the manuscript; H.Z., J.C., and Z.S. revised the manuscript; All authors discussed the results and commented on the manuscript.

## Supporting information



Supporting Information

Supplemental Table 1‐9

## Data Availability

The data that support the findings of this study are available in the supplementary material of this article.

## References

[1] H. Zhang , J. Chen , M. Adams , Arch. Virol. 2001, 146, 2331. 11811683 10.1007/s007050170006

[2] Y. Xu , S. Fu , X. Tao , X. Zhou , Annu. Rev. Phytopathol. 2021, 59, 351. 34077238 10.1146/annurev-phyto-020620-113020

[3] X. Yang , J. Huang , C. Liu , B. Chen , T. Zhang , G. Zhou , Front. Microbiol. 2016, 7, 2140. 28101087 10.3389/fmicb.2016.02140 PMC5210121

[4] M. Yang , A. Ismayil , Y. Liu , Annu. Rev. Virol. 2020, 7, 403. 32530794 10.1146/annurev-virology-010220-054709

[5] S. Zhao , Y. Li , PLoS Pathog. 2021, 17, e1009242. 33630970 10.1371/journal.ppat.1009242 PMC7906326

[6] L. Wang , H. Xie , X. Zheng , J. Chen , S. Zhang , J. Wu , Crop J. 2021, 9, 553.

[7] L. Li , H. Zhang , C. Chen , H. Huang , X. Tan , Z. Wei , J. Li , F. Yan , C. Zhang , J. Chen , Z. Sun , Proc. Natl. Acad. Sci. USA 2021, 118, e2016673118. 33836579 10.1073/pnas.2016673118 PMC7980396

[8] D. Baulcombe , Nature 2004, 431, 356. 15372043 10.1038/nature02874

[9] X. Wu , S. Zou , Z. Chen , Y. Zhang , J. Zhu , N. Ma , J. Tang , C. Chu , X. Pan , Theor. Appl. Genet. 2011, 122, 915. 21140255 10.1007/s00122-010-1498-z

[10] Y. He , H. Zhang , Z. Sun , J. Li , G. Hong , Q. Zhu , X. Zhou , S. MacFarlane , F. Yan , J. Chen , New Phytol. 2017, 214, 388. 27976810 10.1111/nph.14376

[11] Z. Yang , Y. Huang , J. Yang , S. Yao , K. Zhao , D. Wang , Q. Qin , Z. Bian , Y. Li , Y. Lan , T. Zhou , H. Wang , C. Liu , W. Wang , Y. Qi , Z. Xu , Y. Li , Cell Host Microbe 2020, 28, 89. 32504578 10.1016/j.chom.2020.05.001

[12] J. Browse , Annu. Rev. Plant Biol. 2009, 60, 183. 19025383 10.1146/annurev.arplant.043008.092007

[13] C. Wasternack , B. Hause , Ann. Bot. 2013, 111, 1021. 23558912 10.1093/aob/mct067 PMC3662512

[14] Z. Cheng , L. Sun , T. Qi , B. Zhang , W. Peng , Y. Liu , D. Xie , Mol. Plant. 2011, 4, 279. 21242320 10.1093/mp/ssq073

[15] L. Pauwels , G. F. Barbero , J. Geerinck , S. Tilleman , W. Grunewald , A. C. Pérez , J. M. Chico , R. V. Bossche , J. Sewell , E. Gil , G. G. Casado , E. Witters , D. Inzé , J. A. Long , G. D. Jaeger , R. Solano , A. Goossens , Nature 2010, 464, 788. 20360743 10.1038/nature08854 PMC2849182

[16] F. Zhang , J. Yao , J. Ke , L. Zhang , V. Lam , X. Xin , X. E. Zhou , J. Chen , J. Brunzelle , P. R. Griffin , M. Zhou , H. E. Xu , K. Melcher , S. Y. He , Nature 2015, 525, 269. 26258305 10.1038/nature14661 PMC4567411

[17] S. Hu , K. Yu , J. Yan , X. Shan , D. Xie , Mol. Plant. 2023, 16, 23. 36056561 10.1016/j.molp.2022.08.011

[18] R. Chen , H. Jiang , L. Li , Q. Zhai , L. Qi , W. Zhou , X. Liu , H. Li , W. Zheng , J. Sun , C. Li , Plant Cell 2012, 24, 2898. 22822206 10.1105/tpc.112.098277 PMC3426122

[19] S. Song , T. Qi , M. Fan , X. Zhang , H. Gao , H. Huang , D. Wu , H. Gao , D. Xie , PLoS Genet. 2013, 9, e1003653. 23935516 10.1371/journal.pgen.1003653 PMC3723532

[20] C. An , L. Deng , H. Zhai , Y. You , F. Wu , Q. Zhai , A. Goossens , C. Li , Mol. Plant. 2022, 15, 1329. 35780296 10.1016/j.molp.2022.06.014

[21] T. Qi , S. Song , Q. Ren , D. Wu , H. Huang , Y. Chen , M. Fan , W. Peng , C. Ren , D. Xie , Plant Cell 2011, 23, 1795. 21551388 10.1105/tpc.111.083261 PMC3123955

[22] Y. Lu , X. Ye , R. Guo , J. Huang , W. Wang , J. Tang , L. Tan , J. Zhu , C. Chu , Y. Qian , Mol. Plant. 2017, 10, 1242. 28645638 10.1016/j.molp.2017.06.007

[23] M. D. Robinson , D. J. McCarthy , G. K. Smyth , Bioinformatics 2010, 26, 139. 19910308 10.1093/bioinformatics/btp616 PMC2796818

[24] D. Yang , H. Shin , H. Kang , Y. Shang , S. Park , D. Jeong , K. Nam , J. Integr. Plant Biol. 2023, 65, 1226. 36511120 10.1111/jipb.13431

[25] K. Kazan , J. Manners , Mol. Plant. 2013, 6, 686. 23142764 10.1093/mp/sss128

[26] H. Zhang , F. Wang , W. Song , Z. Yang , L. Li , Q. Ma , X. Tan , Z. Wei , Y. Li , J. Li , F. Yan , J. Chen , Z. Sun , Nat. Commun. 2023, 14, 3011. 37230965 10.1038/s41467-023-38805-x PMC10213043

[27] L. Li , H. Zhang , Z. Yang , C. Wang , S. Li , C. Cao , T. Yao , Z. Wei , Y. Li , J. Chen , Z. Sun , Nat. Commun. 2022, 13, 6920. 36376330 10.1038/s41467-022-34649-z PMC9663503

[28] D. Yang , Y. Yang , Z. He , Mol. Plant. 2013, 6, 675. 23589608 10.1093/mp/sst056

[29] M. Alazem , N. S. Lin , Mol. Plant Pathol. 2015, 16, 529. 25220680 10.1111/mpp.12204 PMC6638471

[30] J. Wu , Y. Zhang , F. Li , X. Zhang , J. Ye , T. Wei , Z. Li , X. Tao , F. Cui , X. Wang , L. Zhang , F. Yan , S. Li , Y. Liu , D. Li , X. Zhou , Y. Li , J. Integr. Plant Biol. 2023, 66, 579. 10.1111/jipb.13580 37924266

[31] H. Zhang , L. Li , Y. He , Q. Qin , C. Chen , Z. Wei , X. Tan , K. Xie , R. Zhang , G. Hong , J. Li , J. Li , C. Yan , F. Yan , Y. Li , J. Chen , Z. Sun , Proc. Natl. Acad. Sci. U.S.A. 2020, 117, 9112. 32253321 10.1073/pnas.1918254117 PMC7183187

[32] Y. Wei , Y. Chang , H. Zeng , G. Liu , C. He , H. Shi , J. Pineal Res. 2018, 64, e12454. 10.1111/jpi.12454 29151275

[33] M. Fu , H. Kang , S. Son , S. Kim , K. Nam , Plant Cell Physiol. 2014, 55, 1892. 25189341 10.1093/pcp/pcu118

[34] Y. Hu , T. Zhao , Y. Guo , M. Wang , K. Brachhold , C. Chu , A. Hanson , S. Kumar , R. Lin , W. Long , M. Luo , J. Ma , Y. Miao , S. Nie , Y. Sheng , W. Shi , J. Whelan , Q. Wu , Z. Wu , W. Xie , Y. Yang , C. Zhao , L. Lei , Y. Zhu , Q. Zhang , ModA 2023, 1, 4.

[35] S. Lee , D. Choi , I. Hwang , B. Hwang , Plant Mol. Biol. 2010, 73, 409. 20333442 10.1007/s11103-010-9629-0

[36] C. W. Li , R. C. Su , C. P. Cheng , Sanjaya, S. J. Y. , T. H. Hsieh , T. C. Chao , M. T. Chan , Plant Physiol. 2011, 156, 213. 21398258 10.1104/pp.111.174268 PMC3091068

