# Supplementary material for: Evolutionary‐Distinct Viral Proteins Subvert Rice Broad‐Spectrum Antiviral Immunity Mediated by the RAV15‐MYC2 Module
Source: Adv Sci (Weinh). 2025 Feb 4;12(12):2412835. doi: 10.1002/advs.202412835 (PMC11948057; doi:10.1002/advs.202412835)
Supplement: Supplementary file 1 — Supporting Information [file ADVS-12-2412835-s001.docx]

Supplementary Information

**Evolutionary-Distinct Viral Proteins Subvert Rice Broad-Spectrum Antiviral Immunity Mediated by the RAV15-MYC2 Module**

*Hehong Zhang, Chaorui Huang, Chenfei Gao, Wenkai Yan, Weiqi Song, Xiaodi Hu, Lulu Li, Zhongyan Wei, Yanjun Li, Jianping Chen, and Zongtao Sun**

**Supplementary Figures 1-22**

**
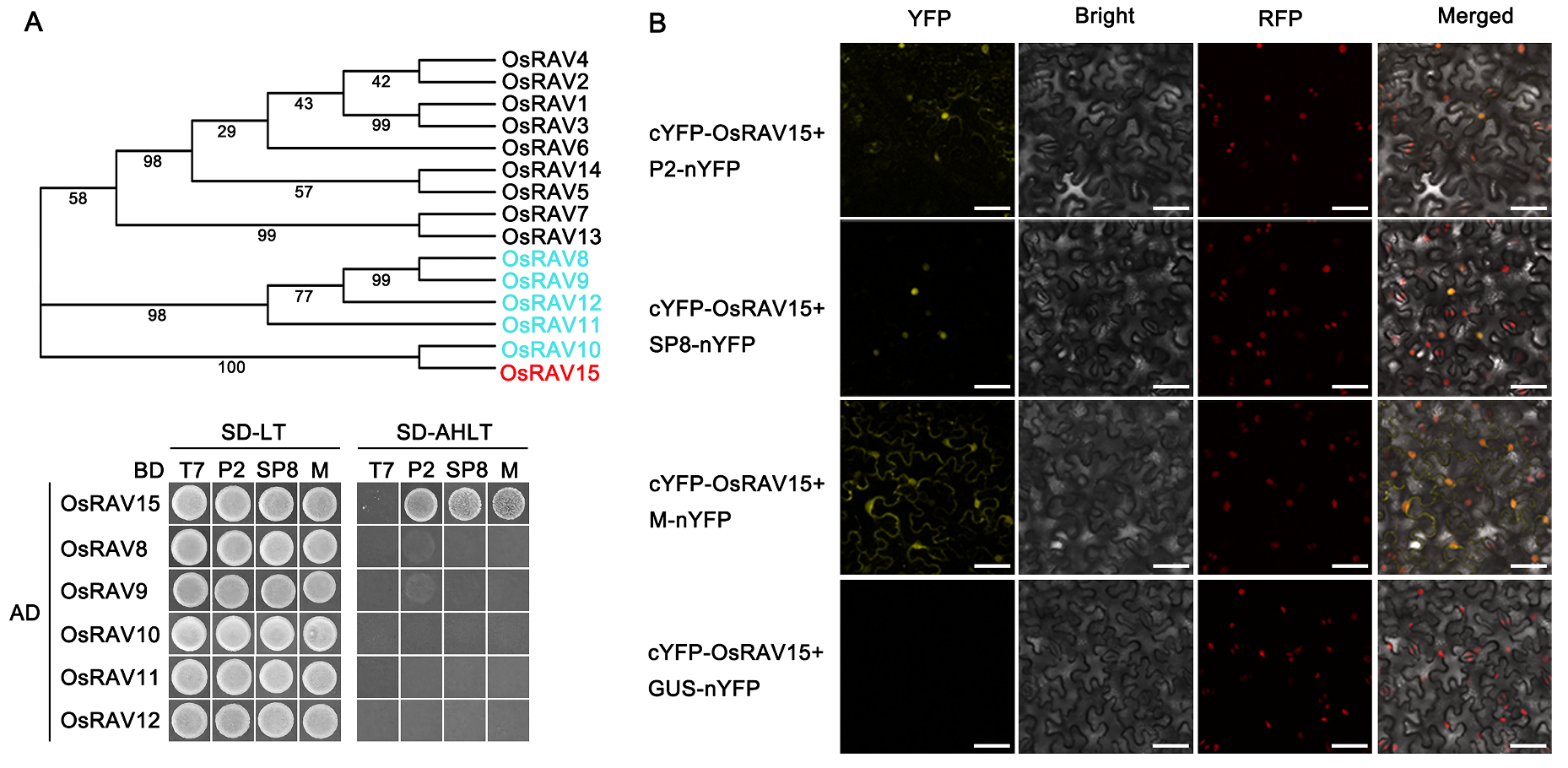
**

**Figure S1.** **OsRAV15 specifically interacts with different viral proteins.** **A.** Y2H assays to test the interaction of different viral proteins with other OsRAVs (OsRAV8, OsRAV9, OsRAV10, OsRAV11 and OsRAV12). **B.** BiFC assays confirming the interactions of OsRAV15 with viral proteins in H2B-RFP transgenic tobaccos. cYFP-OsRAV15 co-expressed with P2/SP8/M-nYFP or the negative controls GUS were injected into *N. benthamiana* leaves. The images were captured by confocal microscopy at 48 h post inoculation (hpi). Scale bar = 50 µm.





**Figure S2.** **The relative expression levels of *OsRAV15, OsMYC2* and *OsJAZ4* genes in transgenic plants.** **A.** The protein levels of OsRAV15 in *OsRAV15* transgenic and NIP rice plants. The protein samples were analyzed by immunoblotting using anti-MYC antibody. CBB staining was used as a loading control to monitor input protein amounts. **B.** The relative expression levels of *OsRAV15* gene in *OsRAV15* and NIP rice plants. **C.** The relative expression levels of *OsRAV15* gene in *OsRAV15-2#/OsMYC2-4#* and NIP rice plants. **D.** The relative expression levels of *OsMYC2* gene in *OsRAV15-2#/OsMYC2-4#* and NIP rice plants. **E.** The relative expression levels of *OsRAV15* gene in *OsRAV15-2#/OsJAZ4* and NIP rice plants. **F.** The relative expression levels of *OsJAZ4* gene in *OsRAV15-2#/OsJAZ4* and NIP rice plants. Values shown are the means ± SD of 3 biological replicates. Significant differences were identified using Tukey's least significant difference tests. * at the top of columns indicates significant difference at p ≤ 0.05.


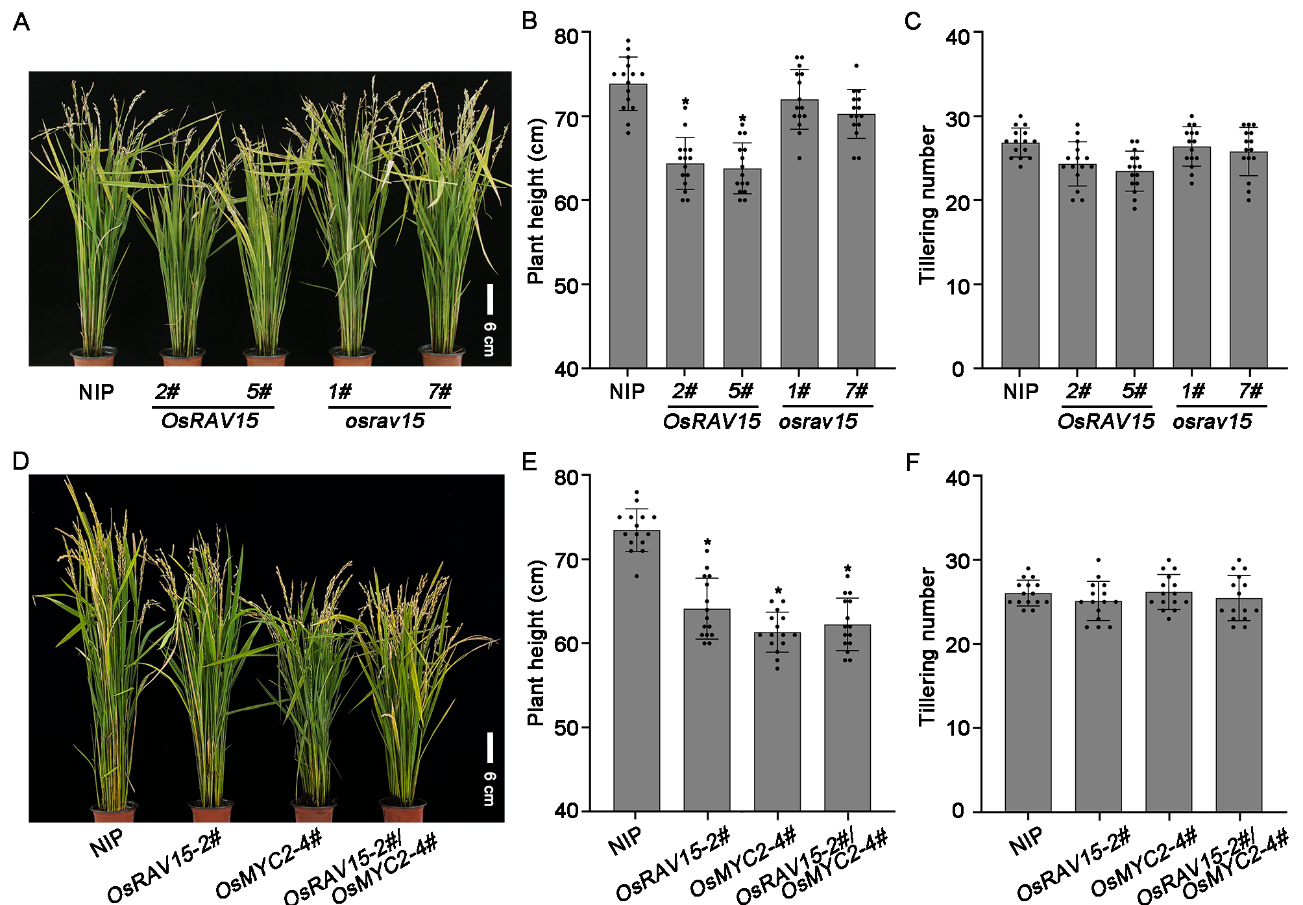


**Figure S3. Phenotypes of *OsRAV15*-related overexpressing transgenic and mutant plants. A.** Phenotypic characteristics of *OsRAV15* overexpressing transgenic and mutant plants in filling period. **D.** Phenotypic characteristics of *OsRAV15-2#/OsMYC2-4#* hybrid plants in filling period. Scale bar = 6 cm. **B** and **E.** Analysis of plant height. **C** and **F.** Analysis of tillering (n = 15). * at the columns indicate significant differences (p ≤ 0.05).


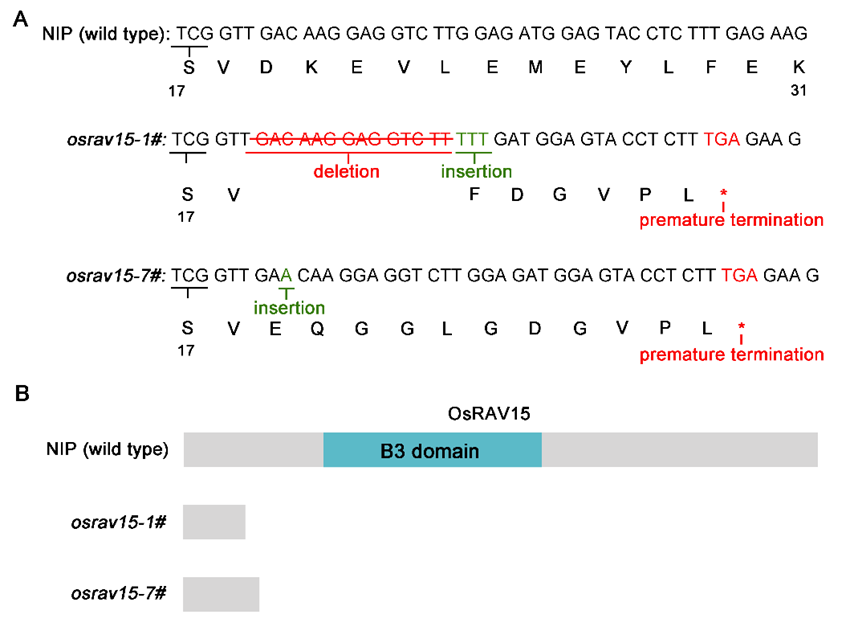


**Figure S4.** **The mutations of *osrav15* rice plants. A.** The mutants of OsRAV15 with the nucleotide deletions shown by red letters and the nucleotide insertion by green letters in the T3 generation. *osrav15-1#* mutant harbored a deletion of 12 nucleotides and a T insertion, which generated a frameshift mutation leading to a premature stop codon. *osrav15-7#* mutant harbored an A insertion, which generated a frameshift mutation leading to a premature stop codon. **B.** Schematic diagram of the full-length and truncated OsRAV15. The B3 domain of OsRAV15 was absent in the mutant *osrav15* rice plants.


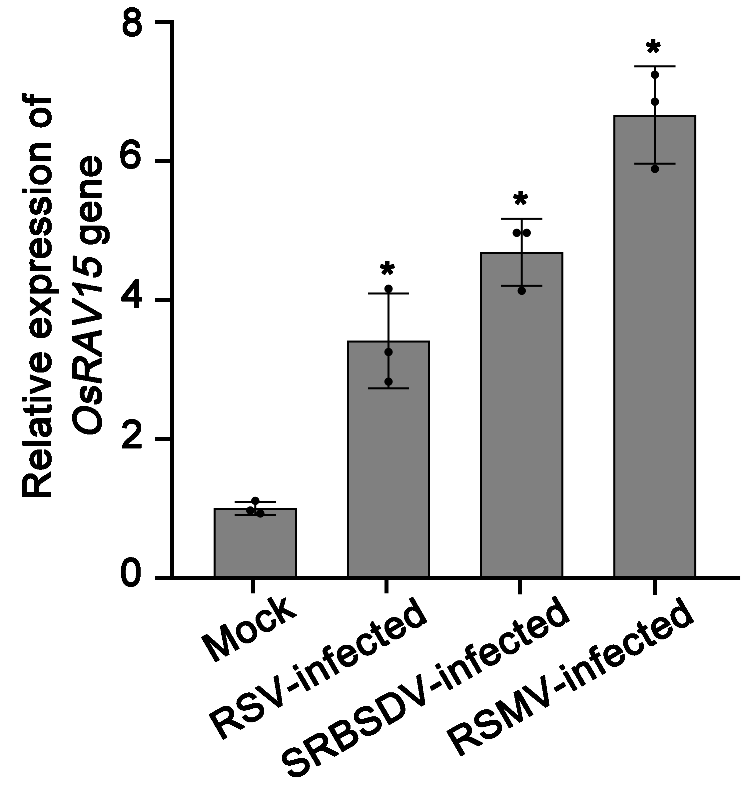


**Figure S5. The relative expression level of the *OsRAV15* gene in RSV/SRBSDV/RSMV-infected and NIP plants at 30 dpi.** Values shown are the means ± SD of 3 biological replicates. Significant differences were identified using Tukey's least significant difference tests. * at the top of columns indicates significant difference at p ≤ 0.05.


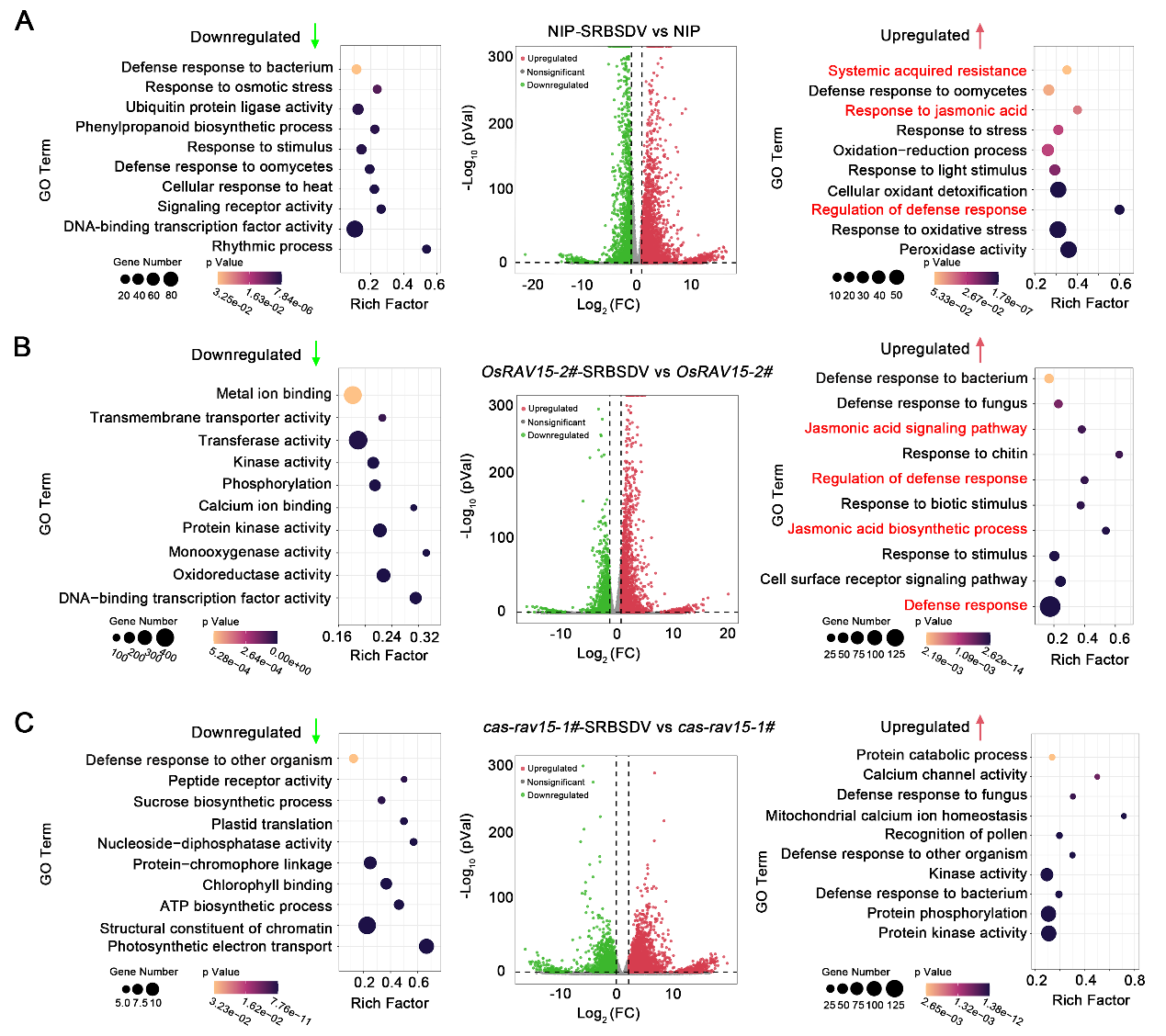


**Figure S6. Transcriptome analysis of *OsRAV15, osrav15* mutant and NIP plants in response to SRBSDV infection. A.** The volcano plot of up/downregulated genes of mock versus SRBSDV in NIP plants (middle). **B.** The volcano plot of up/downregulated genes of mock versus SRBSDV in *OsRAV15-2#* overexpressing plants (middle). **C.** The volcano plot of up/downregulated genes of mock versus SRBSDV in *osrav15-1#* mutant plants (middle). A red dot stands for one up-regulated gene, a green dot for one down-regulated gene and a gray dot stands for a gene with no significant change. All DEGs were selected using cut-off p ≤ 0.05 and fold-change >2 compared with controls. Gene ontology (GO) enrichment analysis of up/downregulated genes (right/left). “Rich factor” shows the ratio between the number of DEGs and the total genes in this pathway.

**
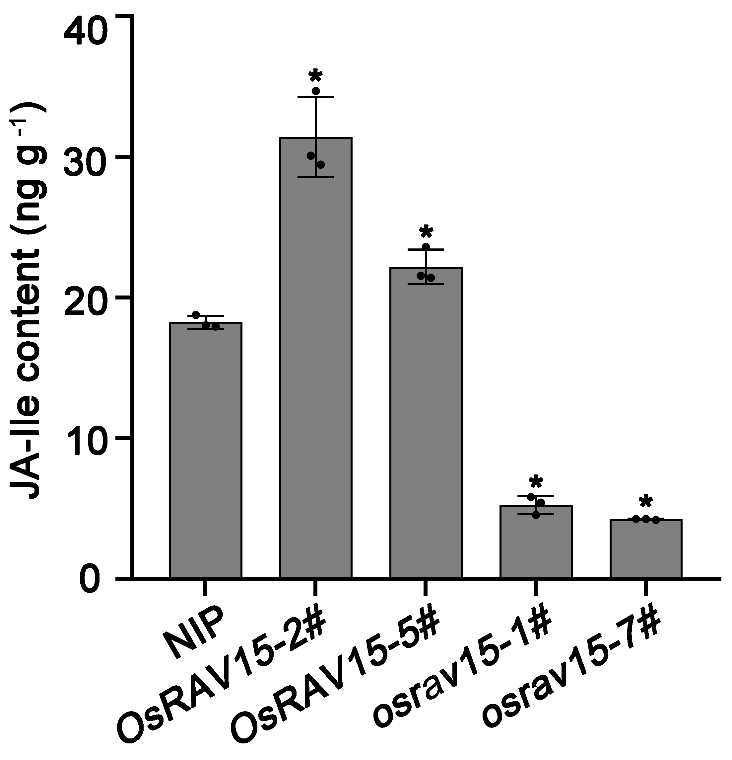
**

**Figure S7. The JA-Ile concentration in *OsRAV15*-overexpressing and *osrav15* mutant plants rice.** Significant differences were identified using Tukey's least significant difference tests. * at the top of columns indicates significant difference at p ≤ 0.05.


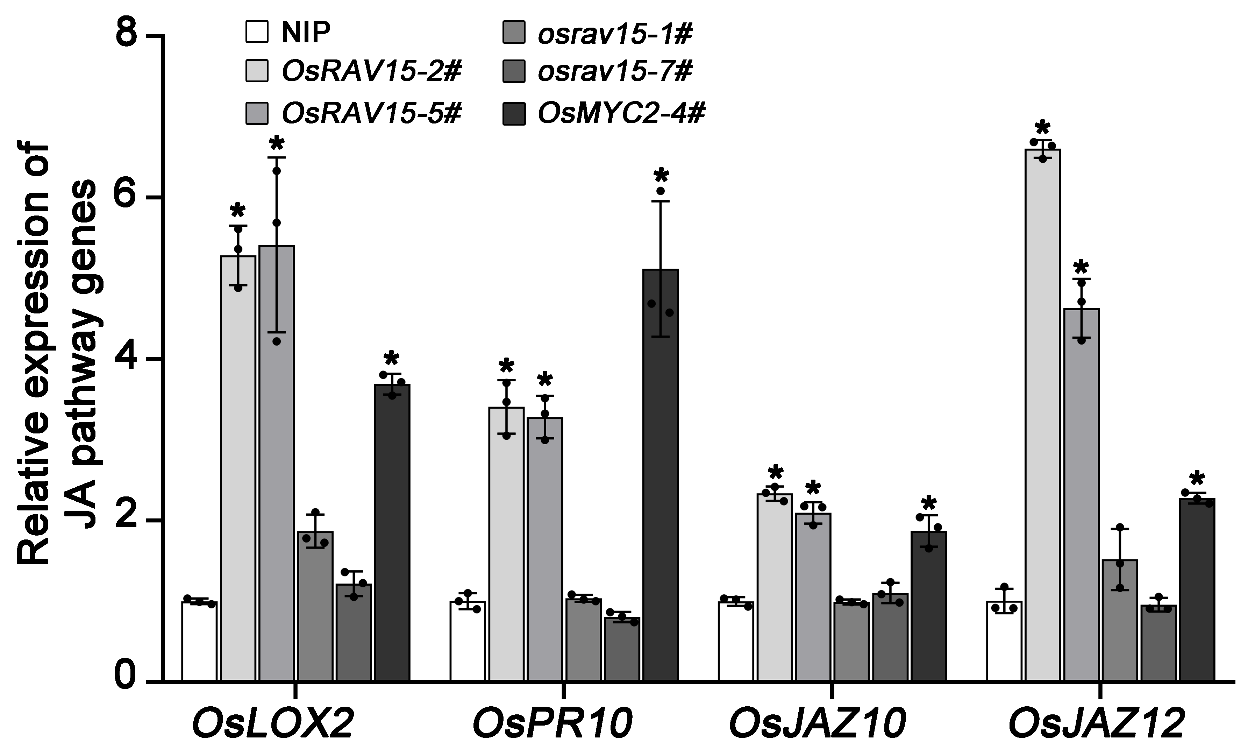


**Figure S8. The relative expression levels of JA pathway genes in** ***OsRAV15/OsMYC2* rice plants.** Values shown are the means ± SD of 3 biological replicates. Significant differences were identified using Tukey's least significant difference tests. * at the top of columns indicates significant difference at p ≤ 0.05.


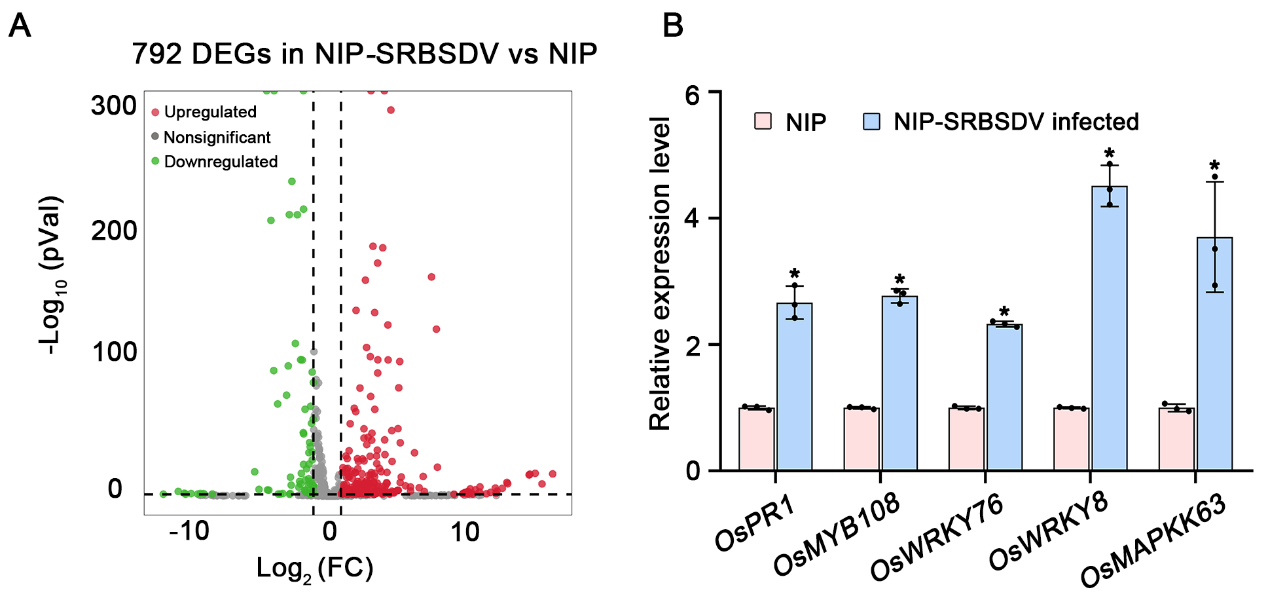


**Figure S9. 792 DEGs regulated by both OsRAV15 and OsMYC2 in NIP plants in response to SRBSDV infection. A.** The volcano plot of up/downregulated genes of mock versus SRBSDV in NIP plants. A red dot stands for one up-regulated gene, a green dot for one down-regulated gene, and a gray dot stands for a gene with no significant change. All DEGs were selected using cut-off p ≤ 0.05 and fold-change >2 compared with controls. **B.** RT-qPCR analyses of *OsPR1, OsMYB108, OsWRKY76, OsWRKY8* and *OsMAPKK63* genes expression levels in mock and SRBSDV-infected NIP plants. Error bars represent SD, values are means ± SD (n = 3 biologically independent replicates per genotype). Significant differences were analyzed using ANOVA followed by Tukey’s multiple comparisons test. * at the columns indicate significant differences (p ≤ 0.05).


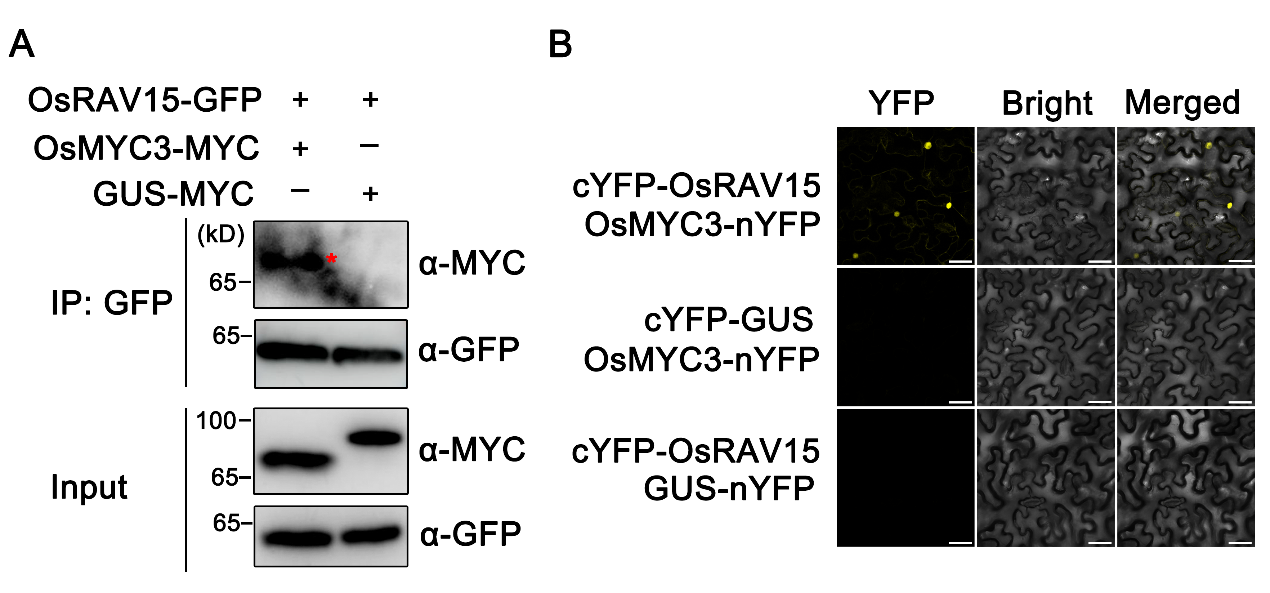


**Figure S10. OsRAV15 directly associated with OsMYC3 transcription factor.** **A.** Co-IP assays indicating that OsRAV15 interacted with OsMYC3 in *N. benthamiana* leaves. OsRAV15-GFP and OsMYC3-MYC or GUS-MYC (negative control) were transiently co-expressed in *N. benthamiana* leaves. Total proteins were extracted, and the supernatant precipitated with GFP beads, followed by Co-IP. The immunoprecipitated (IP) and input proteins were analyzed using anti-GFP and anti-MYC antibodies. The red asterisks represent the specific band. **B.** BiFC assays showing the interactions between OsRAV15 and OsMYC3 in *N. benthamiana* leaves. cYFP-OsRAV15 co-expressed with OsMYC3-nYFP or the negative controls were injected into *N. benthamiana* leaves. The images were captured by confocal microscopy at 48 hpi. Scale bar = 50 µm.

**
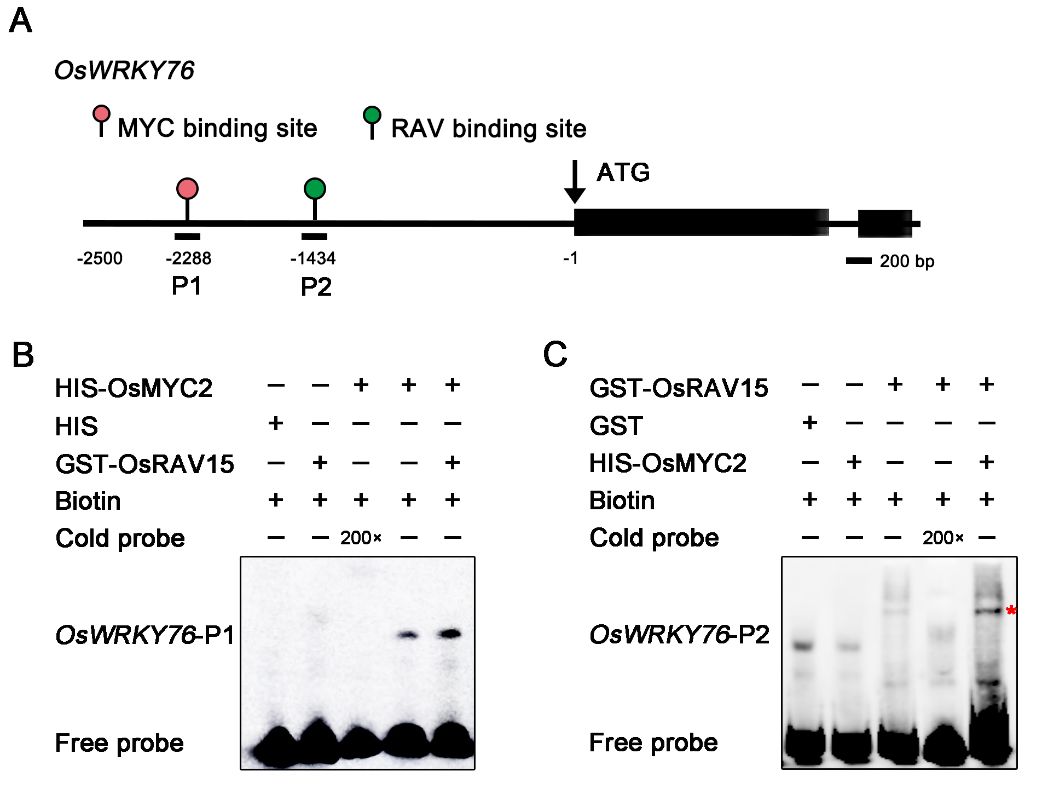
**

**Figure S11. Electrophoretic mobility shift assays (EMSA) showed that OsRAV15 or OsMYC2 all directly bound the promoters of *OsWRKY76*. A.** Schematic diagram of *OsWRKY76* genes with exons displayed as black boxes for EMSA assays. P1 contained MYC binding site; P2 contained RAV binding site. **B.** HIS-OsMYC2 bound specifically to the digoxigenin-labeled MYC2 binding site of *OsWRKY76* promoter. GST-OsRAV15 promoted HIS-OsMYC2 binding to the promoter of *OsWRKY76*. **C.** GST-OsRAV15 bound specifically to the digoxigenin-labeled RAV binding site of *OsWRKY76* promoter. HIS-OsMYC2 promoted GST-OsRAV15 binding to the promoter of *OsWRKY76*. Nonlabeled cold probes were added in 200-fold of the biotinylated native probes. The red asterisks represent the specific band.


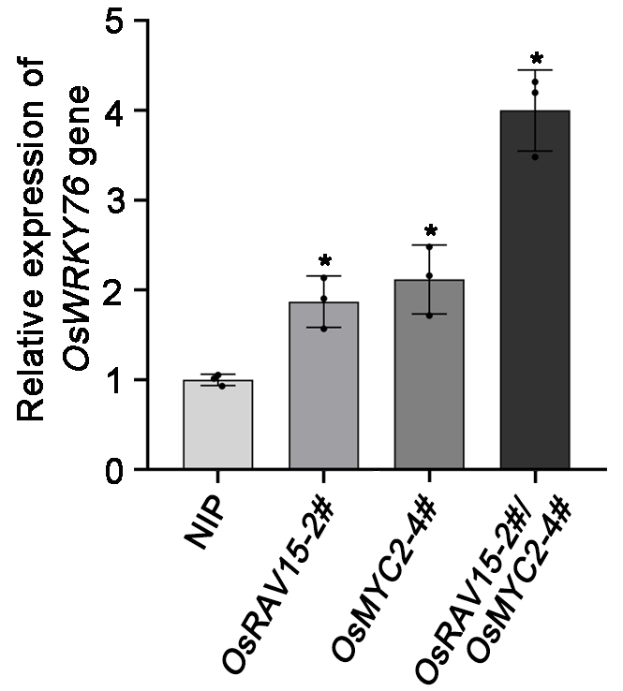


**Figure S12.** **The relative expression levels of *OsWRKY76* gene in *OsRAV15/OsMYC2* rice plants.** Values shown are the means ± SD of 3 biological replicates. Significant differences were identified using Tukey's least significant difference tests. * at the top of columns indicates significant difference at p ≤ 0.05.


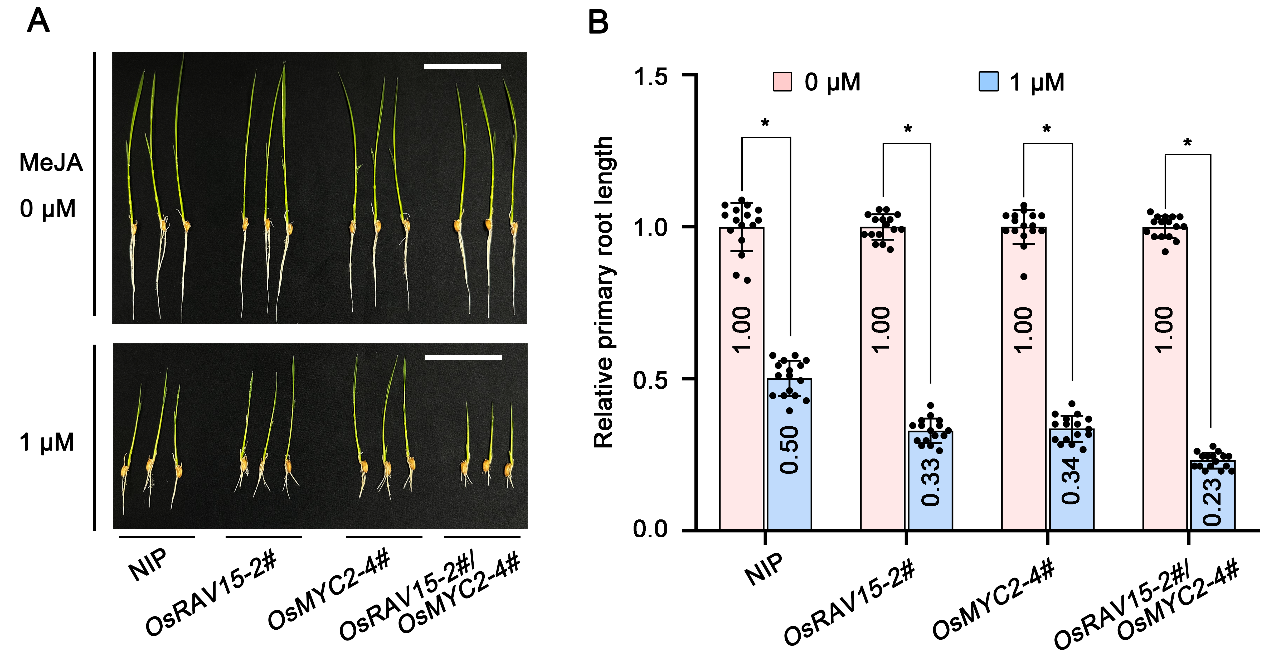


**Figure S13. The effect of JA on the primary root length of *OsRAV15-2#/OsMYC2-4#* hybrid plants.** **A.** Phenotypes of NIP (n=18), *OsRAV15-2#* (n=18)*, OsMYC2-4#* (n=18) and *OsRAV15-2#/OsMYC2-4#* (n=18) seedlings treated with 1 µM MeJA. At least 15 germinated seeds were placed in culture solution containing different concentrations of MeJA for about 5 days, scale bar = 2 cm. **B.** The primary root lengths of NIP, *OsRAV15-2#, OsMYC2-4#* and *OsRAV15-2#/OsMYC2-4#* relative to the control plants. Values were obtained from at least 15 seedlings. * at the top of columns indicates significant difference (p ≤ 0.05).


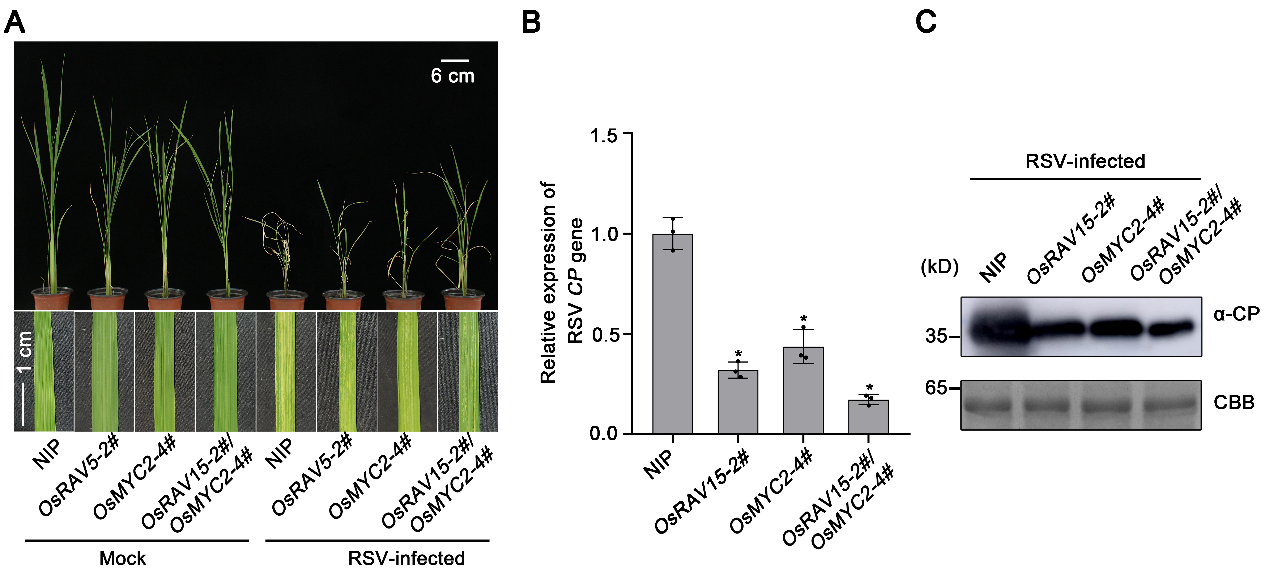


**Figure S14. *OsRAV15-2#/OsMYC2-4#* hybrid plants confer more resistance to RSV infection in rice compared to overexpressing either *OsRAV15-2#* or *OsMYC2-4#* plants.** **A.** Viral symptoms in *OsRAV15-2#* (n=20), *OsMYC2-4#* (n=25), *OsRAV15-2#/OsMYC2-4#* (n=25) transgenic plants and NIP (n=25) in response to RSV infection. The phenotypes were observed and photos taken at 30 dpi. Scale bars = 6 cm. **B.** The relative expression levels of RSV *CP* in RSV-infected *OsRAV15-2#, OsMYC2-4#, OsRAV15-2#/OsMYC2-4#* transgenic plants and NIP rice plants as detected by RT-qPCR at 30 dpi. Error bars represent SD, values are means ± SD (n = 3 biologically independent replicates per genotype). Significant differences were analyzed using ANOVA followed by Tukey’s multiple comparisons test. * at the columns indicate significant differences (p ≤ 0.05). **C.** The accumulation of RSV CP protein in RSV-infected *OsRAV15-2#, OsMYC2-4#, OsRAV15-2#/OsMYC2-4#* and NIP plants by western blotting. CBB serves as the loading control to monitor input protein amounts. And p values of statistic tests (B) were provided in Table S3.

**
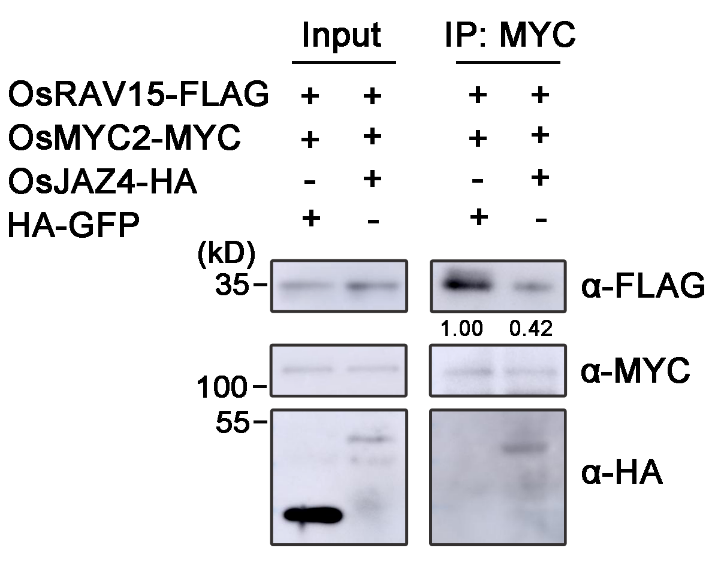
**

**Figure S15.** **Protein competition analyzed by Co-IP assays to confirm OsJAZ4 interfered with the interaction between OsRAV15 and OsMYC2.** OsRAV15-FLAG and OsMYC2-MYC were infiltrated with or without OsJAZ4-HA in leaves of *N. benthamiana,* HA-GFP serves as negative control. The samples were collected at 48 hpi for coimmunoprecipitation with MYC beads. The IP and input proteins were then analyzed using anti-MYC, anti-FLAG and anti-HA antibodies.


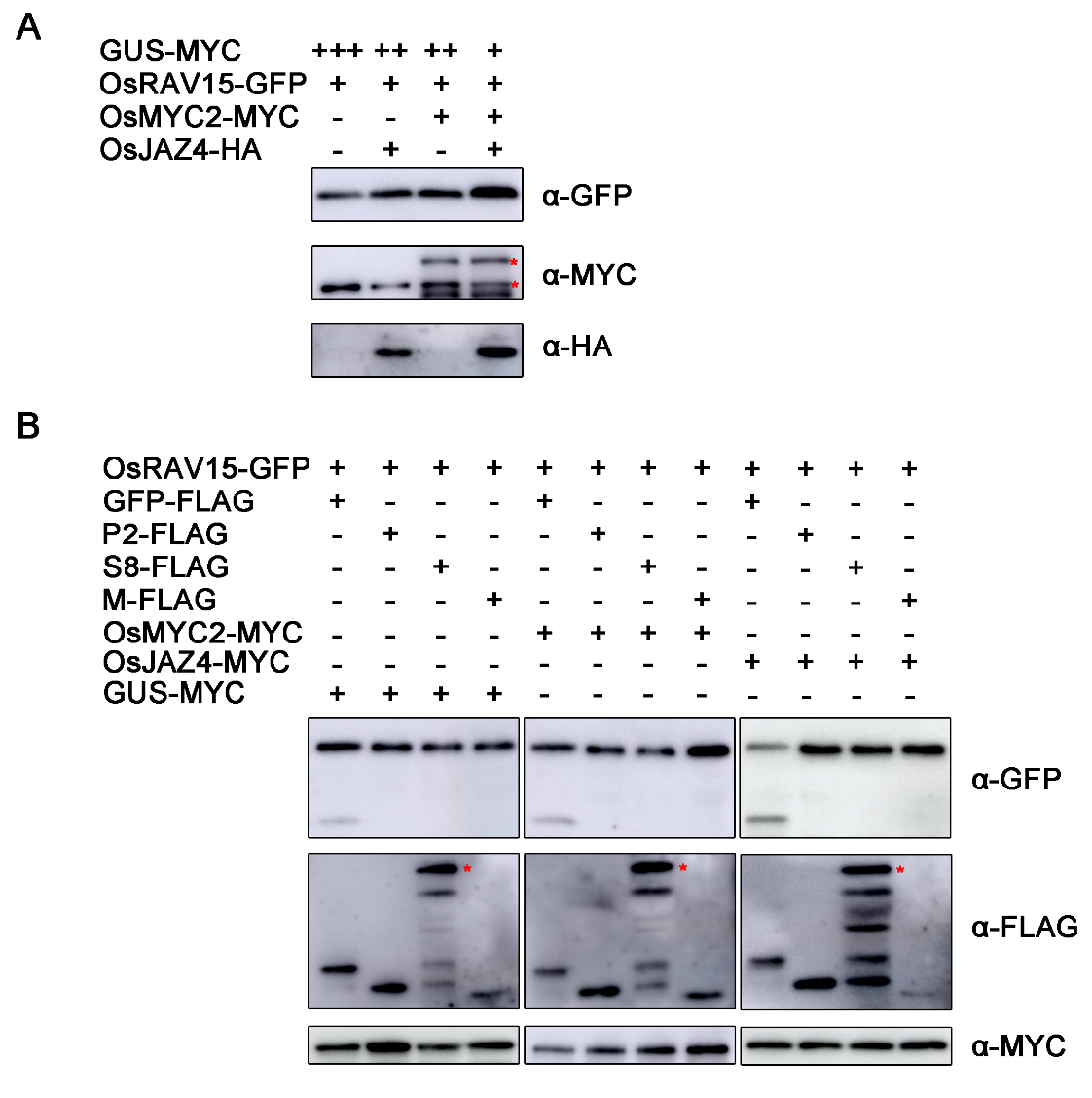


**Figure S16.** **The protein expression in Fig. 5F (A) and Fig. 6E (B).**

**
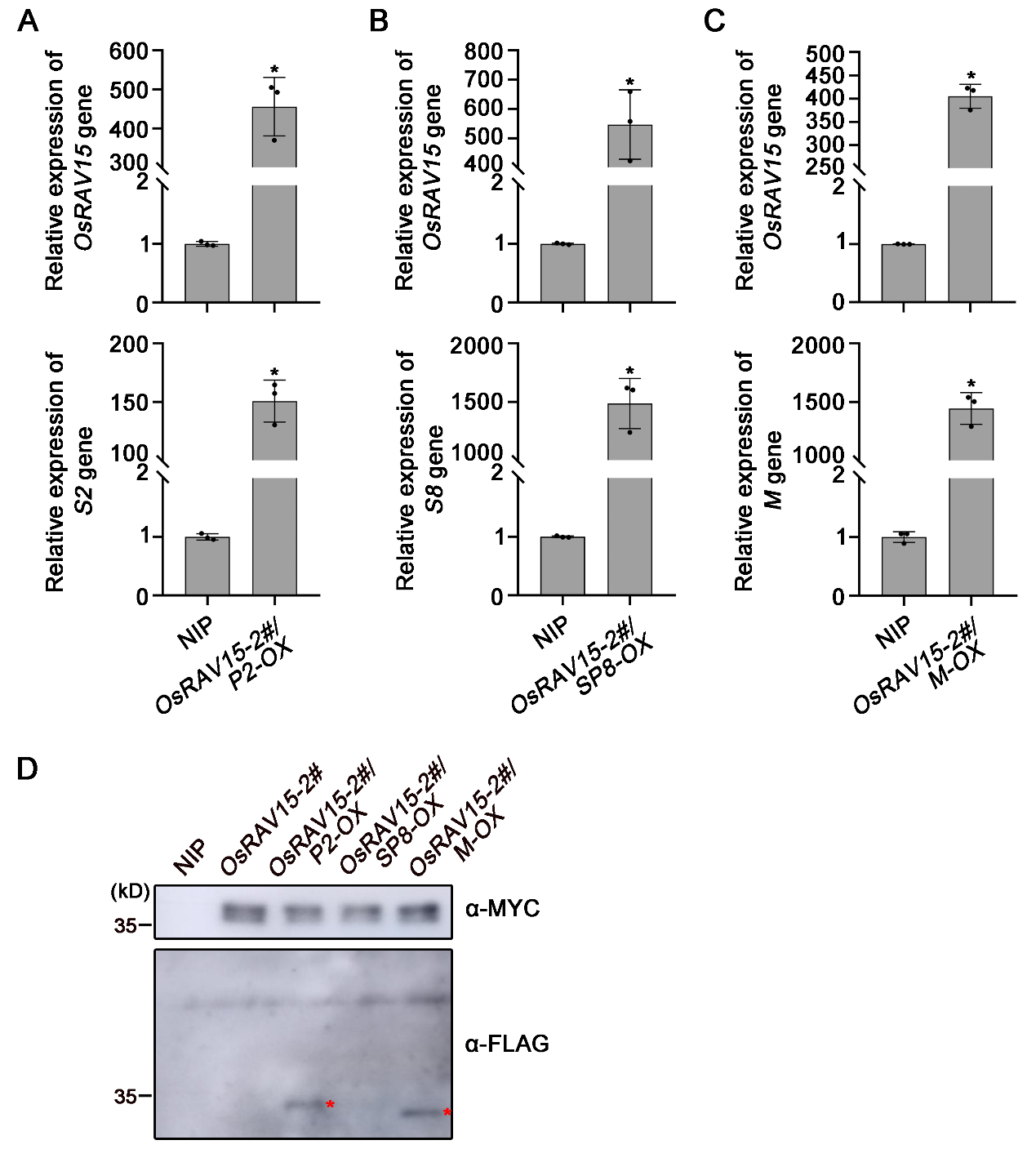
**

**Figure S17.** **The relative expression of the *OsRAV15* gene in OsRAV15-viral protein hybrid plants.** **A.** The relative expression levels of *OsRAV15* and *S2* gene in *OsRAV15-2#/P2-OX* and WT (NIP) rice plants. **B.** The relative expression levels of *OsRAV15* and *S8* gene in *OsRAV15-2#/SP8-OX* and NIP rice plants. **C.** The relative expression levels of *OsRAV15* and *M* gene in *OsRAV15-2#/M-OX* and NIP rice plants. Values shown are the means ± SD of 3 biological replicates. Significant differences were identified using Tukey's least significant difference tests. * at the top of columns indicates significant difference at p ≤ 0.05. **D.** The protein expression detection in hybrid plants by western blotting assays. The protein samples were analyzed by immunoblotting using anti-MYC or anti-FLAG antibody. Since there is no tag in *SP8-OX* plants and the absence of a specific SP8 antibody, we were unable to detect SP8 protein expression in the *OsRAV15-2#/SP8-OX* plants. The red asterisks represent the specific band.

**
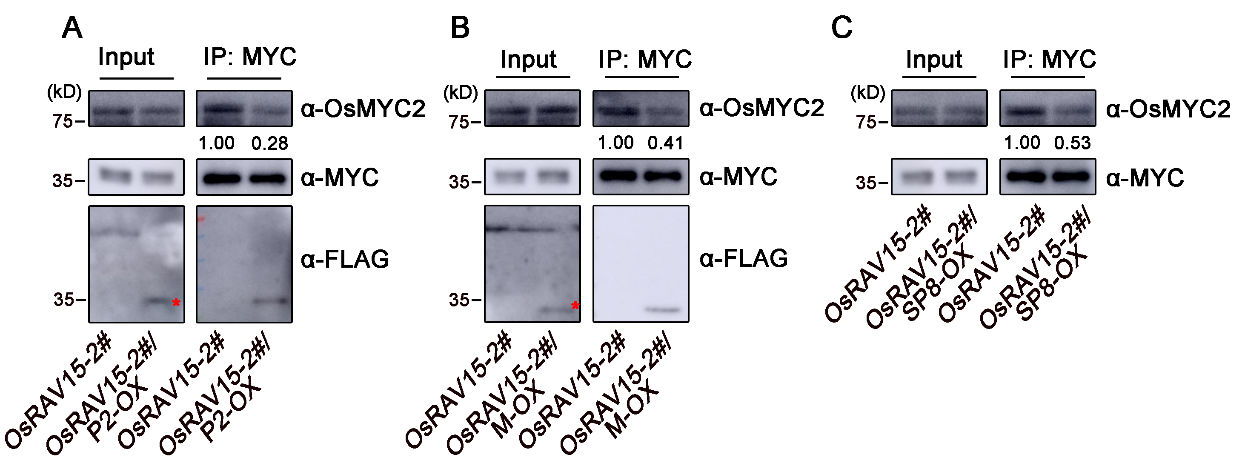
**

**Figure S18.** **Viral proteins inhibit the interaction between OsRAV15 and OsMYC2 in P2/SP8/M-OsRAV15 hybrid transgenic plants.** The samples were coimmunoprecipitated with MYC beads. The IP and input proteins were then analyzed using anti-MYC, anti-FLAG or anti-OsMYC2 antibodies. Since there was no specific SP8 antibody, we could not detect the expression of SP8 protein in *OsRAV15-2#/SP8-OX* plants. The red asterisks represent the specific band.


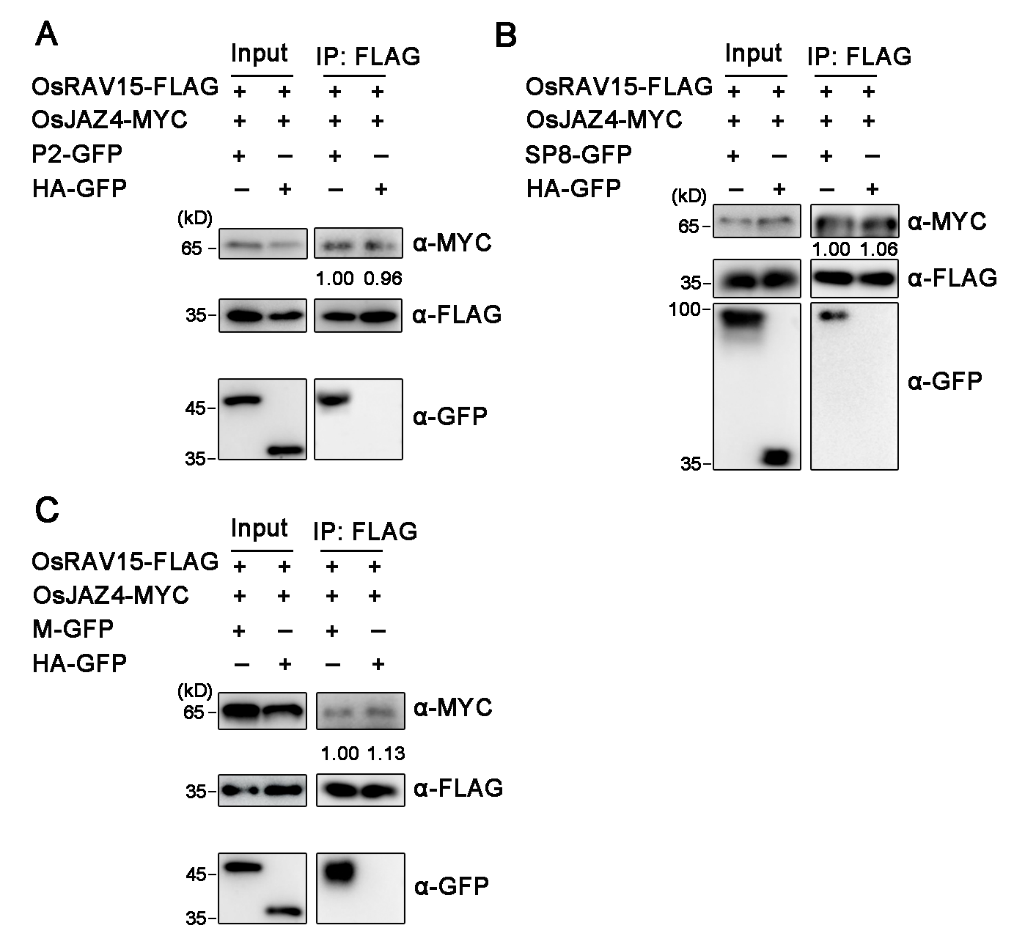


**Figure S19. Protein competition analyzed by Co-IP assays showing that** **viral proteins P2, SP8 and M have no significant influence on the interaction between OsRAV15 and OsJAZ4.** **A, B, C.** OsRAV15-FLAG and OsJAZ4-MYC were infiltrated with or without P2-GFP (**A**), SP8-GFP (**B**) and M-GFP (**C**) in leaves of *N. benthamiana,* HA-GFP serves as negative control. The samples were collected at 48 hpi for coimmunoprecipitation with FLAG beads. The IP and input proteins were then analyzed using anti-MYC, anti-FLAG and anti-GFP antibodies.


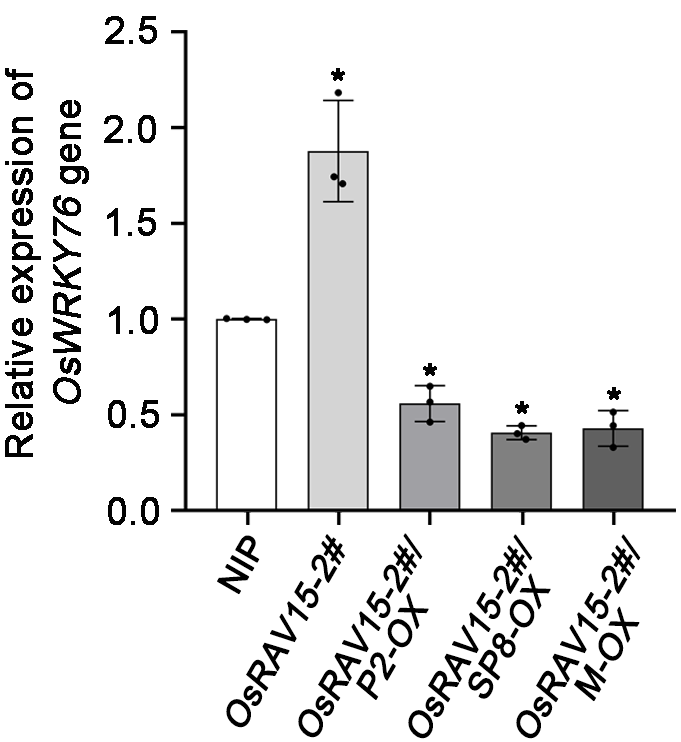


**Figure S20. The relative expression level of the *OsWRKY76* gene in *OsRAV15-2#, OsRAV15-2#/P2-OX, OsRAV15-2#/SP8-OX* and *OsRAV15-2#/M-OX* plants.** Values shown are the means ± SD of 3 biological replicates. Significant differences were identified using Tukey's least significant difference tests. * at the top of columns indicates significant difference at p ≤ 0.05.


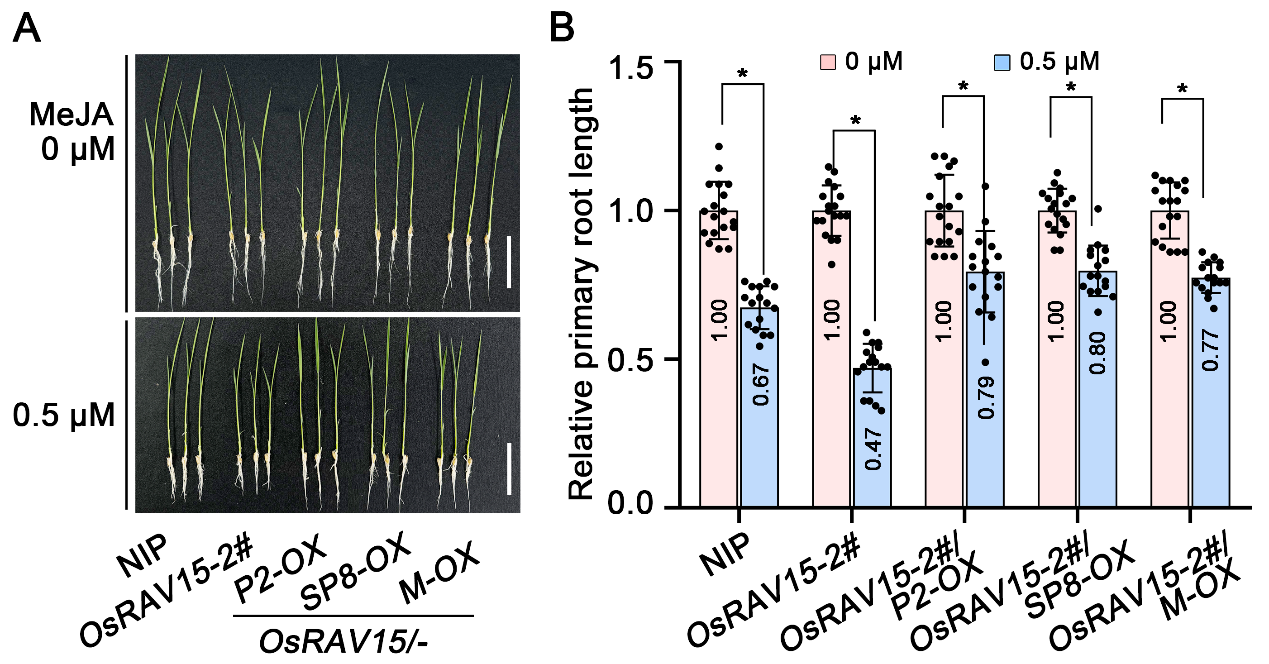


**Figure S21. JA sensitivity assays in OsRAV15 and viral protein hybrid plants.** **A.** Phenotypes of NIP, *OsRAV15-2#,* *OsRAV15-2#/P2-OX*, *OsRAV15-2#/SP8-OX* and *OsRAV15-2#/M-OX* seedlings treated with 0.5 µM MeJA. At least 15 germinated seeds were placed in culture solution containing different concentrations of MeJA for about 5 days, scale bar = 2 cm. **B.** The primary root lengths of NIP, *OsRAV15-2#, OsRAV15-2#/P2-OX*, *OsRAV15-2#/SP8-OX* and *OsRAV15-2#/M-OX* relative to the control plants. Values were obtained from at least 15 seedlings. * at the top of columns indicates significant difference (p ≤ 0.05).

**
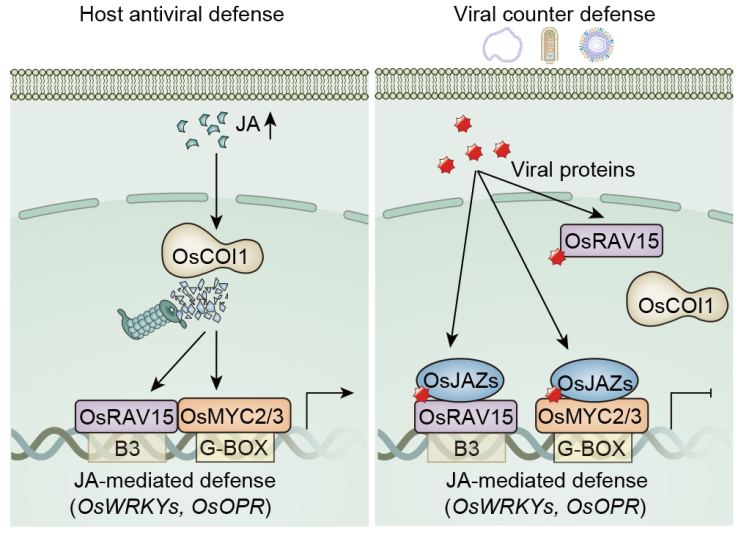
**

**Figure S22. A model describing the role of the identified different viral effectors in disturbing the OsRAV15-mediated broad-spectrum antiviral immunity.** Upon infection of different viruses, JA levels rapidly increased in rice plants, which allow the OsMYC2/3 transcription factors to be released. As a transcriptional activator, OsRAV15, cooperates with OsMYC2/3 to regulate JA-responsive genes involved in antiviral immunity. To counteract the host antiviral immunity, independently evolved viral transcriptional repressors suppress JA-mediated defense by association with OsJAZ4 protein to cooperatively repress the OsRAV15-OsMYC2/3 complex, leading to a compromise of host immunity by attacking OsRAV15-mediated broad-spectrum resistance.
